# Emerging cGAS‐STING Agonist‐Based Nanotherapeutics: Mechanistic Insights and Applications in Cancer Combination Therapy

**DOI:** 10.1002/advs.202509890

**Published:** 2025-08-21

**Authors:** Zhaomeng Wang, Yongjun Wang, Zhonggui He, Caigang Liu

**Affiliations:** ^1^ Department of Oncology Innovative Cancer Drug Research and Engineering Center of Liaoning Province Cancer Stem Cell and Translation Medicine Lab Shengjing Hospital of China Medical University Shenyang Liaoning 110004 China; ^2^ Wuya College of Innovation Shenyang Pharmaceutical University Shenyang Liaoning 110016 China

**Keywords:** cancer immunotherapy, cGAS‐STING agonists, combination therapies, mechanisms, nanotherapeutics

## Abstract

The cyclic guanosine monophosphate‐adenosine monophosphate synthase (cGAS)‐stimulator of interferon genes (STING) pathway has emerged as a promising target for cancer immunotherapy. Activation of the cGAS‐STING pathway holds significant potential to enhance antitumor immunity. However, their efficacy remains constrained by various drug delivery and pharmacological obstacles, including poor stability, limited cellular uptake, inefficient intracellular delivery, suboptimal tumor targeting, and immunotoxicity. There is a growing focus on the design and application of nanoparticulate drug delivery systems (nano‐DDSs) to improve the delivery of cGAS‐STING agonists for safe, effective, and specific targeting. This review outlines the latest advancements in cGAS‐STING agonist‐based nanotherapeutics. First, the research background of the cGAS‐STING pathway and nanotechnology is briefly outlined to emphasize the promise of STING agonist‐based nanotherapeutics in cancer immunotherapy. Second, the progress in the development of cGAS‐STING agonists and recent advancements in cGAS‐STING agonist‐based nanotherapeutics are overviewed. Moreover, the molecular mechanisms and applications of STING agonist‐based combination therapies are discussed, including synergistic strategies with chemotherapy, radiotherapy, immunotherapy (e.g., immune checkpoint blockade (ICB), indoleamine 2,3‐dioxygenase (IDO) inhibitor, cancer vaccine, adoptive cell therapy (ACT)), phototherapy, sonodynamic therapy (SDT), and targeted therapy. Finally, the challenges and future perspectives of STING agonist‐based nanotherapeutics in clinical cancer therapy are proposed.

## Graphical abstract

This review summarizes recent advances in cGAS‐STING agonist‐based nanotherapeutics for cancer combination therapy. It explores nanoparticle‐mediated delivery strategies, underlying molecular mechanisms, and combinatorial applications with chemotherapy, radiotherapy, targeted therapy, phototherapy, sonodynamic therapy, and immunotherapy. Key translational challenges and future directions are discussed to facilitate the clinical development of next‐generation STING‐targeted immunotherapies.

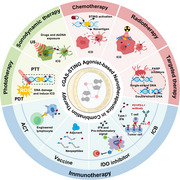

## 1 Introduction

Cancer immunotherapy has revolutionized oncology, offering transformative therapeutic options for a wide range of malignancies. Despite remarkable progress, significant challenges remain in achieving broader clinical applicability and sustained therapeutic efficacy.^[^ ^1^ ^]^ Therefore, the development of safe, potent, and clinically viable immunotherapeutic strategies remains an urgent unmet medical need in cancer treatment.

Among emerging immunotherapeutic targets, the cGAS‐STING pathway garnered significant attention due to its intrinsic role in initiating and amplifying endogenous immune responses.^[^ ^2^ ^]^ This pathway plays a crucial role in innate immune detection by recognizing double‐stranded DNA (dsDNA), whether derived from pathogens or the host, and converting it into a potent second messenger, 2′,3′‐cyclic GMP‐AMP (cGAMP).^[^ ^3^ ^]^ Upon binding to STING, a transmembrane protein localized in the endoplasmic reticulum, cGAMP induces allosteric activation of STING, triggering its translocation to the Golgi apparatus. This, in turn, triggers the recruitment and activation of TANK‐binding kinase 1 (TBK1), which then phosphorylates key transcription factors, including interferon regulatory factor 3 (IRF3) and nuclear factor‐kappa B (NF‐κB).^[^ ^4^ ^]^ Activation of these pathways culminates in the production and secretion of type I interferons (IFN‐I) and other proinflammatory cytokines, which play a pivotal role in the propagation of cancer immunity.

**Figure 1:**
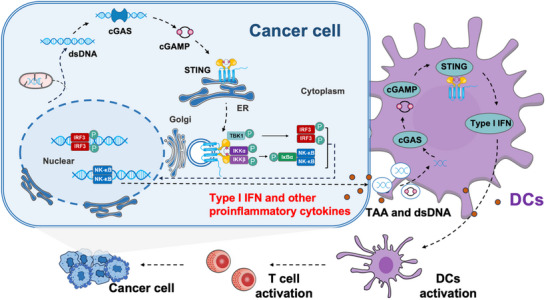
cGAS‐STING pathway activation and its role in antitumor immunity. cGAS senses cytoplasmic DNA and catalyzes the synthesis of cGAMP, which activates STING and triggers the TBK1‐IRF3 signaling cascade and the production of IFN‐I in tumor cells. IFN‐I activates DCs, which capture and present tumor‐associated antigens (TAAs) from tumor cells, ultimately stimulating TAA‐specific CTLs. Additionally, DNA fragments from dying tumor cells can activate cGAS in DCs, further enhancing IFN‐I production and amplifying the immune response.

IFN‐I bridges the innate and adaptive immune responses, promoting the maturation of antigen‐presenting cells (APCs), particularly dendritic cells (DCs). This process improves the APCs’ capacity to present tumor antigens to naïve T cells, thereby priming and activating tumor antigen‐specific CD8+ cytotoxic T lymphocytes (CTLs), enabling them to infiltrate tumors and exert cytotoxic effects (**Figure** **1**).^[^ ^5^ ^]^ Moreover, STING activation facilitates the recruitment and activation of additional effector immune cells, including natural killer (NK) cells and neutrophils, further strengthening antitumor immunity.^[^ ^6^ ^]^ Additionally, IFN‐I contributes to reprogramming tumor‐associated macrophages (TAMs), shifting them from an immunosuppressive M2 phenotype to a pro‐inflammatory M1 phenotype,^[^ ^2^, ^7^ ^]^ boosting tumor immunogenicity, and amplifying antitumor immune responses.

Beyond its intrinsic role in tumor immunity, the STING pathway plays a fundamental role in potentiating standard cancer therapies. Accumulating evidence suggests that many conventional cancer treatments, including DNA‐damaging chemotherapy and radiotherapy, elicit additional therapeutic benefits by iatrogenic activation of the STING pathway.^[^ ^8^ ^]^ These insights have fueled intense interest in developing STING agonists as a promising strategy for cancer immunotherapy. Indeed, preclinical studies have consistently demonstrated that STING agonists can potently elicit antitumor immunity in a variety of cancer types. Encouraged by these promising findings, numerous clinical trials have been initiated to evaluate the therapeutic potential of STING agonists, many of which are still ongoing.^[^ ^9^ ^]^ However, despite significant preclinical success, no STING agonist has yet achieved pharmaceutical approval. Multiple barriers hinder the clinical translation of STING agonists, including poor stability, short half‐life, inefficient cytosolic delivery, off‐target effects, drug resistance, and unexpected immunotoxicities.^[^ ^2^, ^9^, ^10^ ^]^ These limitations collectively compromise therapeutic efficacy and restrict their widespread clinical implementation. Thus, innovative and efficient delivery strategies are highly warranted to overcome these hurdles and unlock the full clinical potential of cGAS‐STING agonists.

Nanotechnology has been extensively explored to develop advanced nano‐DDSs for cancer treatment, offering significant advantages for improving drug availability and ensuring precisely targeted delivery.^[^ ^11^ ^]^ First, nano‐DDSs provide an effective strategy to improve the pharmacological properties of drug payloads, such as improving solubility, in vivo stability, pharmacokinetics, and biodistribution profiles while preventing biologics from premature release and degradation.^[^ ^12^ ^]^ The limited solubility and low membrane permeability of STING agonists can be effectively addressed by incorporating them into nano‐DDSs. Second, nano‐DDSs can be engineered with stimuli‐responsive functionalities to optimize intracellular delivery and enable tumor‐selective drug release or activation. These systems can respond to specific biochemical changes in the tumor microenvironment (TME), such as hypoxia, pH, redox potential, or enzymes, as well as external stimuli including light, magnetic fields, or electrical signals.^[^ ^13^ ^]^ This precise control over drug activation and release enhances therapeutic efficacy while minimizing off‐target effects and immune‐related toxicities, which is particularly crucial for potent STING agonists known to cause severe dose‐limiting toxicities. Third, nano‐DDSs enable surface modification by targeting ligands such as peptides, antibodies, and glycans, facilitating enhanced delivery of STING pathway agonists to tumors, lymphoid organs, and specific cell populations.^[^ ^14^ ^]^ This targeted approach can improve therapeutic precision, maximize efficiency, and minimize off‐target effects. Finally, nano‐DDSs provide a versatile platform for the co‐delivery of multiple therapeutic agents within a single nanocarrier at defined ratios.^[^ ^15^ ^]^ This strategy facilitates synergistic interactions between STING agonists and other therapies, such as chemotherapeutics, radiotherapy sensitizers, immune checkpoint inhibitors, and photosensitizers, thereby broadening the therapeutic window and enhancing the potential for combined cancer treatments. Thus, nanotechnology plays a pivotal role in optimizing the delivery of STING agonists to maximize therapeutic efficacy, minimize side effects, and offer a powerful platform for the development of advanced combination cancer therapies.

**Figure 2:**
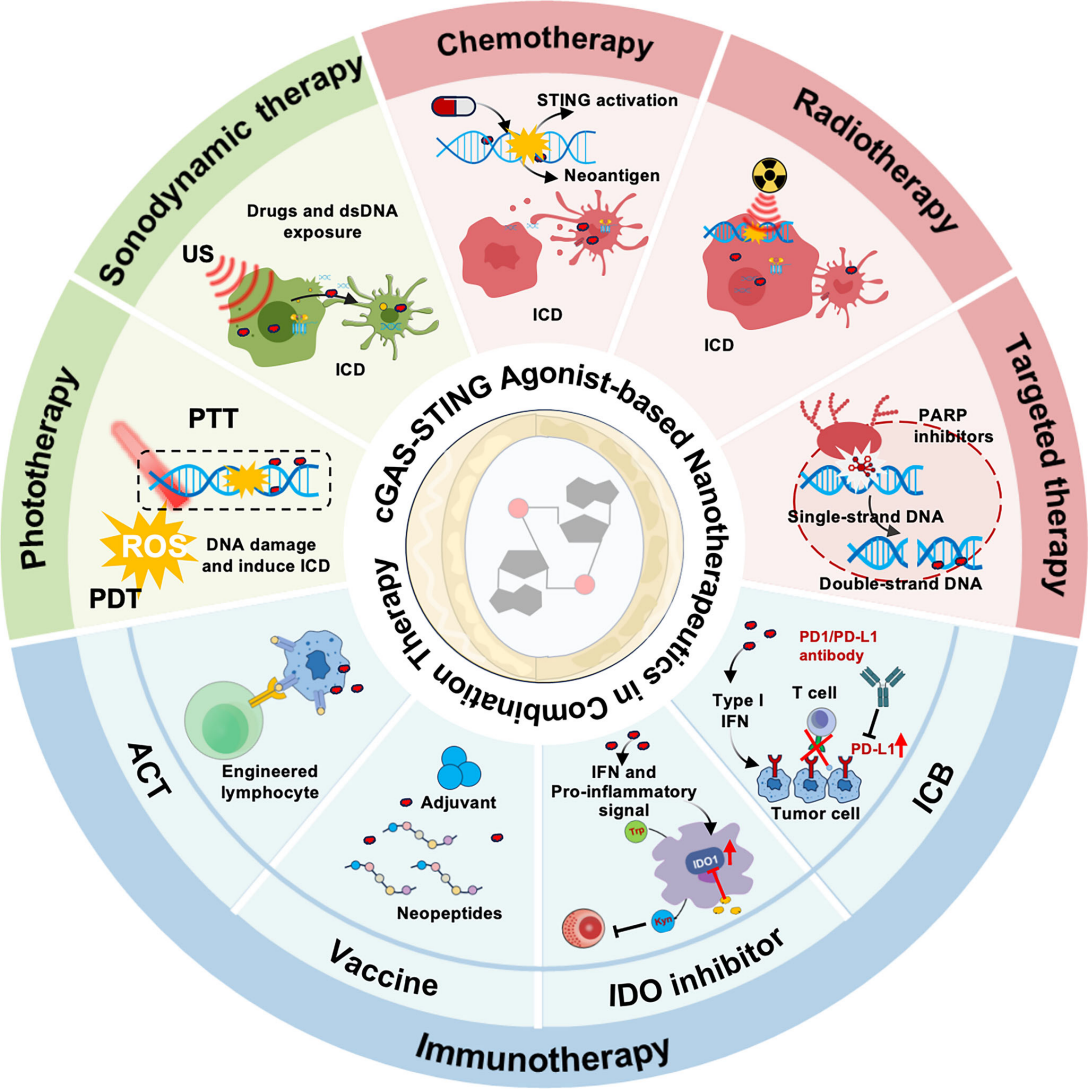
Schematic representation of cGAS‐STING pathway agonist‐based nanotherapeutics, highlighting their molecular mechanisms, therapeutic rationale, and applications in combination strategies for cancer immunotherapy.

STING agonist‐based nanotherapeutics have recently gained increasing attention in cancer immunotherapy, owing to the efficient antitumor efficacy achieved through the integration of STING agonists with nanotechnology. As a research hotspot in cancer immunotherapy, the cGAS‐STING pathway has been extensively reviewed, focusing on its biological mechanisms, types of modulators, and the design of nano‐DDSs for STING agonists in cancer therapy.^[^ ^4^, ^9^, ^16^ ^]^ However, the rationale and applications of STING agonist‐based nanotherapeutics in combination with other cancer therapies remain underexplored. This review provides a comprehensive overview of the current development and clinical progress of cGAS‐STING agonists, highlights emerging nanotherapeutics strategies, and elucidates their molecular mechanisms and rationale within distinct combinatorial therapy contexts. Furthermore, we discuss recent advancements in STING agonist‐based nanotherapeutics in combination therapies, evaluate their therapeutic applications (**Figure** **2**), and offer perspectives on future clinical translation to optimize the efficacy and safety of these promising immunotherapeutic approaches.

## 2 Development and Emerging Nanotherapeutics of cGAS‐STING Agonists for Cancer Immunotherapy

### 2.1 Development of cGAS‐STING Agonists in Cancer Immunotherapy

**Figure 3:**
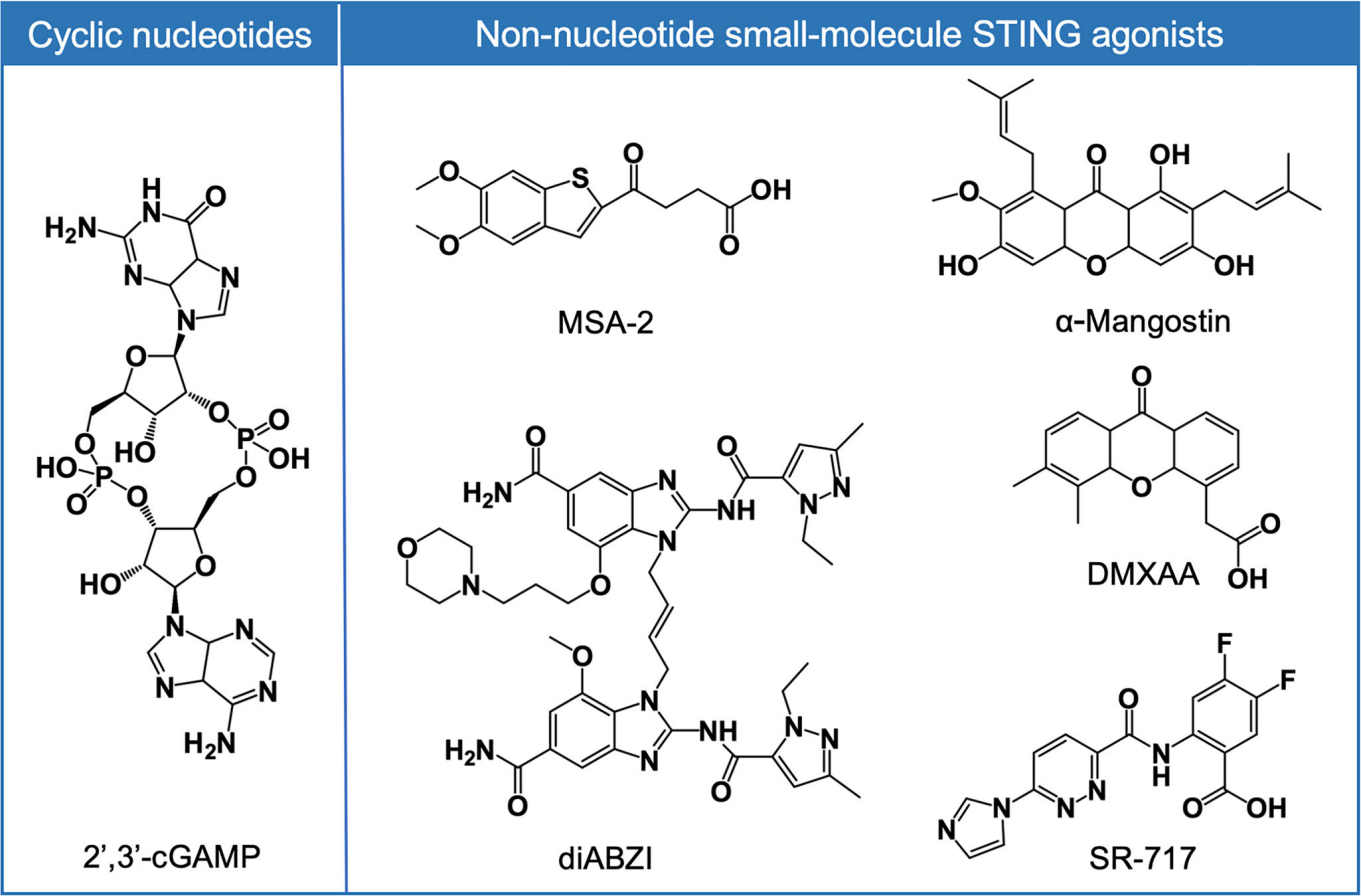
Representative chemical structures of cyclic dinucleotides and different non‐nucleotide small‐molecule STING agonists.

Recent advances in understanding the mechanistic basis of cGAS‐STING activation have significantly accelerated the development of therapeutic agents targeting this pathway. Currently, the therapeutic potential of STING agonists, particularly for cancer treatment, has been validated in both preclinical and clinical settings, showing substantial therapeutic promise for stimulating antitumor immune responses. STING agonists are classified into two categories: cyclic dinucleotides (CDNs) and non‐CDN small molecules (**Figure** **3**).

Natural CDNs, including 2′,3′‐cyclic guanosine monophosphate‐adenosine monophosphate (2′,3′‐cGAMP), 3′,3′‐cyclic guanosine monophosphate‐adenosine monophosphate (3′,3′‐cGAMP), cyclic di‐adenosine monophosphate (c‐di‐AMP),^[^ ^17^ ^]^ and cyclic di‐guanosine monophosphate (c‐di‐GMP),^[^ ^18^ ^]^ differ in their biochemical properties, which influence their interaction with the STING receptor.^[^ ^19^ ^]^ Among these, 2′,3′‐cGAMP is synthesized by cGAS in mammalian cells in response to cytosolic DNA detection, while others are produced by bacterial cGAS and serve as signaling molecules in prokaryotic systems. However, natural CDNs exhibit inherently poor drug‐like properties, particularly owing to inefficient intracellular delivery and susceptibility to hydrolysis by ectonucleotide pyrophosphatase/phosphodiesterase 1.^[^ ^20^ ^]^

To overcome these limitations, second‐generation synthetic CDNs have been developed with enhanced membrane permeability and resistance to enzymatic degradation. These compounds, with improved drug‐like properties, are advancing through the pharmaceutical development pipeline. For instance, ADU‐S100 (Rp, Rp‐2′,3′‐c‐di‐AMPSS) is a dithio‐substituted cyclic diadenine featuring mixed phosphorothioate linkages, designed to improve stability and lipophilicity, thus promoting STING activation.^[^ ^21^ ^]^ Despite these advancements, both natural and synthetic CDNs still suffer from poor pharmacokinetic profiles and limited cellular uptake, primarily due to the highly hydrophilic and negatively charged nature of their backbone.

The limitations of natural STING agonists have spurred recent research and therapeutic development toward non‐nucleotide small molecules with improved cellular uptake and enzymatic stability. Notably, non‐CDN STING agonists, such as 5,6‐dimethylxanthenone‐4‐acetic acid (DMXAA), dimeric amidobenzimidazole (diABZI), benzothiophene oxobutanoic acid (MSA‐2), and SR‐717, have demonstrated the capacity to elicit systemic antitumor immunity across various cancer types. Among these, DMXAA, initially identified as a potent vascular‐disrupting agent, was later recognized as a strong STING agonist in mice.^[^ ^22^ ^]^ It exhibited robust antitumor effects in melanoma, breast cancer, and fibrosarcoma models, with tumor suppression highly dependent on the STING pathway and CD8+ T cells.^[^ ^23^ ^]^ This promising STING‐driven antitumor activity led to its advancement into clinical trials. However, despite its efficacy in preclinical models, DMXAA ultimately failed in a Phase III clinical trial for non‐small cell lung cancer due to insufficient efficacy.^[^ ^24^ ^]^ The failure was primarily attributed to species‐specific variations in the STING protein, which prevented DMXAA from binding human STING (hSTING) isoforms, despite high sequence and structural similarity to mouse STING (mSTING).^[^ ^25^ ^]^ In response to this limitation, alternative derivatives have been developed, such as α‐mangostin, a xanthone derivative, which has demonstrated stronger binding affinity for hSTING than for mSTING.^[^ ^26^ ^]^

Building upon the development of non‐CDN STING agonists, amidobenzimidazole (ABZI)‐based compounds have emerged as another promising class, demonstrating the ability to bind both mSTING and hSTING, as recently reported by Ramanjulu and colleagues at GlaxoSmithKline.^[^ ^27^ ^]^ diABZI exhibited over 400‐fold greater potency than cGAMP in activating STING and inducing IFN production, with an EC_50_ of 130 nM in human peripheral blood mononuclear cells (PBMCs) expressing wild‐type STING. Notably, diABZIs were also effective in human PBMCs with other STING isoforms (e.g., R232H/R232H, HAQ/HAQ) as well as in murine PBMCs, facilitating preclinical evaluation of its pharmacological characteristics and anticancer effects in murine tumor models. In a CT26 colorectal tumor model, intravenous administration of diABZI led to complete tumor suppression in 80% of treated mice and significantly prolonged overall survival. This finding established diABZI as the first non‐CDN small‐molecule STING agonist capable of binding both mSTING and hSTING, activating antitumor immunity, and inhibiting tumor growth following intravenous administration. This represents a major advancement in the field of cancer immunotherapy based on STING activation.

In parallel, MSA‐2, identified through a high‐throughput, cell‐based phenotypic screening of millions of compounds by Addona and colleagues at Merck, emerged as another highly selective STING agonist with superior cell permeability.^[^ ^28^ ^]^ Designed as a prodrug to activate STING, MSA‐2 undergoes reversible, noncovalent dimerization in acidic pH environments, with the dimer (rather than the monomer) serving as the pharmacologically active ligand. This distinctive activation mechanism sets MSA‐2 apart from conventional covalent STING agonists, highlighting its innovative design. Importantly, MSA‐2 demonstrated tumor‐targeting activity in response to the acidic TME, with significantly higher accumulation in subcutaneous MC38 tumors relative to plasma and non‐tumor tissues. This tumor‐selective enrichment enhanced the production ofIFN‐β and proinflammatory cytokines at the tumor site, resulting in complete regression in 80–100% of treated mice via subcutaneous, intratumoral, and oral administration routes. Furthermore, MSA‐2 induced durable antitumor immunity and demonstrated strong synergy with anti‐PD‐1 in multiple tumor models, including B16‐F10 melanoma, CT26 colorectal, MC38 colorectal, and LL‐2 lung cancer, reinforcing its potential as a clinically relevant STING‐mediated immunotherapy.

Expanding the repertoire of non‐nucleotide STING agonists, SR‐717 has emerged as a promising candidate with potential for both systemic and oral administration, thereby enhancing the therapeutic options available for STING‐based cancer immunotherapy.^[^ ^29^ ^]^ Mechanistically, SR‐717 activates STING in a manner similar to CDNs, wherein two SR‐717 molecules bind at the base of the intersubunit cleft of the STING dimer, triggering a closed lid conformation similar to the binding mode of 2′3’‐cGAMP. Notably, SR‐717 exhibits broad affinity for all major human hSTING variants as well as mSTING, while also demonstrating enhanced stability under physiological conditions. These properties enable detailed in vivo investigations of its pharmacological profile and therapeutic potential in mouse tumor models. Importantly, intraperitoneal injection of SR‐717 at therapeutic doses led to elevated plasma IFN production and robust STING‐mediated antitumor responses in syngeneic mouse models of MC38 colorectal adenocarcinoma and B16‐F10 melanoma.

Beyond the non‐CDN small‐molecule STING agonists discussed above, which exhibit favorable drug‐like properties and enhanced cytosolic delivery compared to the anionic and highly water‐soluble CDNs, alternative STING activators have also been identified for their potential in tumor immunotherapy. These include metal ions (e.g., Mn, Zn, Co),^[^ ^30^ ^]^ cytosolic dsDNA,^[^ ^31^ ^]^ DNA‐damaging agents,^[^ ^32^ ^]^ and polymer‐based materials such as PC7A.^[^ ^33^ ^]^ A summary of the clinical development status of all STING agonists in cancer immunotherapy is presented in **Table** **1**.

**Table 1:** Clinical trials of STING agonists for cancer therapy.

STING agonist	Route of administration	Tumor type	Treatment regimen	Trial phase	Status	Clinical trial code
MK‐1454	Intratumoral	Advanced solid tumors or lymphomas	Monotherapy or in combination with pembrolizumab	Phase I	Completed	NCT03010176
MK‐1454	Intratumoral	Recurrent head and neck squamous cell carcinoma	MK‐1454 + pembrolizumab	Phase II	Completed	NCT04220866
ADU‐S100 (MIW815)	Intratumoral	Advanced solid tumors or lymphoma	Monotherapy or in combination with spartalizumab	Phase I	Terminated	NCT03172936
ADU‐S100 (MIW815)	Intratumoral	Head and neck cancer	MIW815 + pembrolizumab	Phase II	Terminated	NCT03937141
ADU‐S100 (MIW815)	Intratumoral	Advanced/metastatic solid tumors or lymphomas	MIW815 + lpilimumab	Phase I	Terminated	NCT02675439
SB 11285	Intravenous	Advanced solid tumors	Monotherapy or in combination with atezolizumab	Phase I/II	Completed	NCT04096638
GSK3745417	Intratumoral	Advanced solid tumors	Monotherapy or in combination with pembrolizumab	Phase I	Active, not recruiting	NCT03843359
E7766	Intratumoral	Non‐muscle invasive bladder cancer	Monotherapy	Phase I	Terminated	NCT04144140
E7766	Intravesical	Non‐muscle invasive bladder cancer	Monotherapy	Phase I	Withdrawn	NCT04109092
MK‐2118	Intratumoral	Advanced solid tumors	Monotherapy or in combination with pembrolizumab	Phase I	Terminated	NCT03249792
SNX281	Intratumoral	Advanced solid tumors	Monotherapy	Phase I	Terminated	NCT04609579
SR‐717	Oral	Advanced solid tumors	Monotherapy	Phase I	Completed	NCT04937153
TAK‐676	Intravenous	Advanced solid tumors	Monotherapy or in combination with pembrolizumab	Phase I	Completed	NCT04879849
BMS‐986301	Intratumoral	Advanced solid tumors	Monotherapy or in combination with nivolumab	Phase I/II	Completed	NCT03956680
exoSTING (CDK 002)	Intratumoral	Advanced/metastatic, recurrent, injectable solid tumors	Monotherapy	Phase I/II	Completed	NCT04592484
SYNB1891	Intratumoral	Advanced solid tumors and lymphoma	SYNB1891 + atezolizumab	Phase I	Terminated	NCT04167137
BI1387446	Intratumoral	Advanced or metastatic csolid tumors	BI1387446 + ezabenlimab	Phase I	Completed	NCT04147234
Dazostinag	Intravenous	Advanced or metastatic solid tumors	Montherapy or in combination with pembrolizumab	Phase I/II	Recruiting	NCT04420884
CRD3874‐SI	Intravenous	Relapsed/refractory acute myeloid leukemia	Monotherapy	Phase I	Recruiting	NCT06626633
CRD3874‐SI	Intravenous	Solid tumors	Monotherapy	Phase I	Recruiting	NCT06021626
IMSA101	Intravenous	Advanced solid tumors	Monotherapy or in combination with nivolumab	Phase I	Completed	NCT04020185
IMSA101	Intratumoral	Metastatic kidney cancer (SPARK)	Personalized ultra‐fractionated stereotactic adaptive radiotherapy (PULSAR) in combination with IMSA101	Phase II	Not yet recruiting	NCT06601296
IMSA101	Intratumoral	Oligoprogressive solid tumor malignancies	PULSAR + pembrolizumab or nivolumab administered with or without IMSA101	Phase II	Terminated	NCT05846659
IMSA101	Intratumoral	Oligometastatic non‐small cell lung cancer and renal cell carcinoma	PULSAR + pembrolizumab or nivolumab administered with or without IMSA101	Phase II	Terminated	NCT05846646
TAK‐500	Intravenous	Select locally advanced or metastatic solid tumors	TAK‐500 with or without pembrolizumab	Phase I/II	Recruiting	NCT05070247

Despite promising preclinical results demonstrating robust antitumor activity, CDNs and other STING agonists have exhibited limited efficacy in clinical trials, both as monotherapies and in combination with ICB. This limited success is largely attributed to poor pharmacokinetics, inadequate tumor antigen presentation, and uncoordinated immune activation.^[^ ^2^ ^]^ Moreover, issues such as nonspecific tissue distribution and dose‐limiting toxicity have dramatically impeded their broader clinical application. A key limitation of current STING agonists is their lack of cell‐ and tissue‐specific targeting, as STING is broadly expressed across immune cells, non‐immune cells (such as endothelial cells), and cancer cells,^[^ ^34^ ^]^ each of which may exhibit distinct responses to STING activation, ultimately influencing therapeutic outcomes and increasing the risk of immune‐related adverse events. Furthermore, while local intratumoral injection of STING agonists has shown potential, its requirement for frequent dosing over several months poses practical challenges for patient adherence, with up to 48% of patients not adhering to the treatment regimen.^[^ ^35^ ^]^ Additionally, repeated intratumoral administration can disrupt the TME and vascular networks, which may inadvertently increase the risk of metastasis.^[^ ^35^, ^36^ ^]^

To overcome these limitations, recent advancements in drug delivery technologies and administration strategies have focused on enhancing the tumor‐specific targeting of STING agonists. By leveraging differential receptor expression and optimizing administration approaches, these strategies aim to improve therapeutic efficacy while minimizing systemic toxicity, potentially addressing key barriers to the clinical translation of STING‐mediated immunotherapy.

### 2.2 Emerging Nanotherapeutics of cGAS‐STING Agonists for Cancer Immunotherapy

Recently, substantial efforts have focused on the development of multifunctional nano‐DDSs to overcome the delivery challenges associated with the in vivo application of cGAS‐STING agonists. A variety of candidate delivery nanoplatforms, such as those based on polymers, liposomes, and inorganic nanoparticles, have been extensively explored to enhance both the local and systemic therapeutic outcomes of cGAS‐STING agonists. As several comprehensive reviews have already summarized the advancements in nano‐DDSs for STING agonists,^[^ ^2^, ^9^, ^16^, ^23^ ^]^ this section highlights key recent advancements in STING agonist‐based nanotherapeutics for cancer immunotherapy.

Lipid‐based nano‐DDSs have demonstrated significant promise in delivering cGAS‐STING agonists, primarily due to their ability to encapsulate both hydrophobic and hydrophilic compounds. Among these, liposomes stand out as one of the most successful lipid‐based nanocarriers, with several commercially available formulations, such as Doxil, Onivyde, and Vyxeos.^[^ ^37^ ^]^ The unique structure of liposomes, with an aqueous core and a lipid bilayer, enables the encapsulation of hydrophilic cargo within the core, while hydrophobic cGAS‐STING agonists can be integrated into the bilayer.

**Figure 4:**
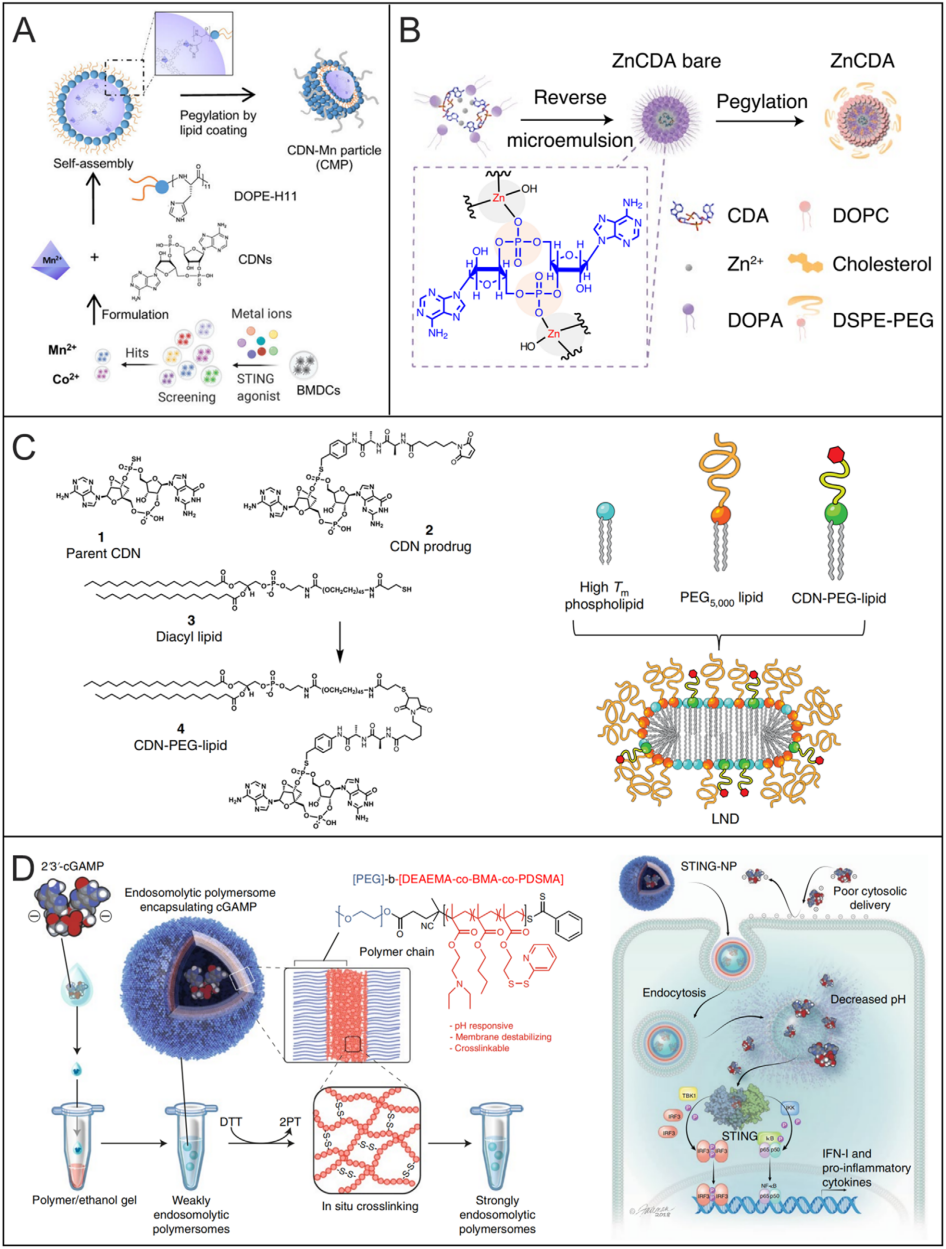
Lipidic and polymeric cGAS‐STING pathway agonist delivery systems for cancer immunotherapy. A) Formulation of CDNs and metal ions for enhanced STING activation. The CMP is composed of CDNs, Mn^2+^, phospholipid‐(histidine)_11_ (DOPE‐H_11_), and a PEG‐lipid layer (DOPC:cholesterol:DSPE‐PEG5000). Mn^2+^ potentiates the IFN‐I activities of STING agonists. Mn^2+^ and CDNs self‐assemble into a coordination polymer. The CDN‐Mn^2+^ coordination polymer is coated with DOPE‐H_11_ through Mn‐histidine coordination to form CDN‐Mn@DOPE, followed by PEGylation with a PEG‐lipid layer, resulting in the formation of the CMPs. Reproduced with permission.^[^ ^38^ ^]^ Copyright 2021, Springer Nature. B) Formulation strategy and preparation of ZnCDA for STING activation. Reproduced with permission.^[^ ^30a^ ^]^ Copyright 2022, Springer Nature. C) Design of nanoparticles for STING agonist delivery. left, Chemical structures of the parent CDN STING agonist (1), CDN prodrug (2), diacyl lipid (3), and CDN‐PEG‐lipid (4). right, Schematic of LND containing high melting temperature phospholipids, PEG5000‐lipids, and CDN‐PEG‐lipid. Adapted with permission.^[^ ^39^ ^]^ Copyright 2022, Springer Nature. D) Schematic of the chemical structure, formulation preparation, and intracellular delivery mechanism of STING‐NPs. Reproduced with permission. ^[^ ^40^ ^]^ Copyright 2019, Springer Nature.

To enhance the efficacy of STING activation, Moon et al. designed a lipid‐based coordination nanoparticle for synergistic cancer metalloimmunotherapy (**Figure** **4**A).^[^ ^38^ ^]^ In this study, metal ions like Mn^2+^ and Co^2+^ were found to function as STING activators that work synergistically with STING agonists to amplify immune responses. Given the essential role of Mn^2+^ in immune function, the authors utilized Mn^2+^coordinated with c‐di‐AMP to self‐assemble into nanoparticles (CND‐Mn^2+^, CMP). These nanoparticles were then complexed with dioleoyl‐sn‐glycero‐3‐phosphoethanolamine‐N‐(histidine)_11_ (DOPE‐H_11_) to form a hydrophobic core (termed CDA‐Mn@DOPE).^[^ ^38^ ^]^ To further enhance stability and prolong circulation, the nanocore was coated with a layer of PEG‐lipid comprising 1,2‐dioleoyl‐sn‐glycero‐3‐phosphocholine (DOPC), 1,2‐distearoyl‐sn‐glycero‐3‐phosphoethanolamine (DSPE)‐PEG5000, and cholesterol, forming a lipidic nanoparticle named CMP_CDA_. Upon intratumoral or systemic intravenous administration, CMP_CDA_ effectively triggered robust antitumor immunity, resulting in significant therapeutic efficacy in multiple murine tumor models, including colorectal cancer, melanoma, and squamous cell carcinoma.^[^ ^38^ ^]^

Similarly, Weichselbaum et al. developed zinc cyclic di‐AMP nanoparticles (ZnCDA) for tumor‐targeted STING activation.^[^ ^30a^ ^]^ ZnCDA was synthesized in two steps: first, the CDA‐loaded Zn phosphate core was generated via coordination polymerization of sodium phosphate, Zn(NO_3_)_2_, and CDA using a reverse microemulsion method. This core was subsequently coated with 1,2‐dioleoyl‐sn‐glycero‐3‐phosphate (DOPA), resulting in monodisperse ZnCDA nanoparticles. The final formulation was further encapsulated within a lipid bilayer composed of cholesterol, DOPC, and DSPE‐PEG2000, enhancing its stability and circulation time (Figure 4B). Upon intravenous administration, ZnCDA extended CDA circulation, facilitated efficient tumor targeting, and elicited potent antitumor responses in various preclinical models. Mechanistically, ZnCDA promoted tumor accumulation by disrupting endothelial cells in the tumor vasculature and selectively targeted TAMs, thereby enhancing antigen processing and presentation to activate antitumor T‐cell responses.^[^ ^30a^ ^]^

Beyond traditional liposomal carriers, Irvine et al. introduced an advanced nanoplatform for systemic cGAMP delivery, designed to enhance tumor penetration and boost anticancer immunotherapy efficacy (Figure 4C).^[^ ^39^ ^]^ In this approach, a CDN prodrug with a dialanine peptide linker was synthesized and covalently conjugated to thiol‐terminated PEGylated lipids through thiol‐maleimide coupling chemistry. The resulting CDN‐PEG5000‐lipids were then assembled with PEG5000‐lipids and high‐melting‐point phospholipids to form lipid nanodiscs (LNDs) with a mean diameter of approximately 26 nm. Compared to conventional liposomes, LNDs demonstrated superior tumor penetration and facilitated effective intracellular delivery of STING agonists.^[^ ^39^ ^]^ Notably, a single dose of LND‐CDNs was sufficient to activate STING and induce robust antitumor immunity across various cancer models, including the aggressive orthotopic 4T1 breast cancer and MC38 colorectal cancer models. Additionally, LNDs elicited long‐term immune memory, preventing tumor rechallenge, while exhibiting minimal systemic toxicity.^[^ ^39^ ^]^ This strategy offers several advantages over conventional liposomal STING agonist delivery, including enhanced tumor penetration and an improved safety profile, ultimately maximizing therapeutic efficacy while minimizing off‐target toxicity.

Apart from lipid‐based carriers, polymeric carriers provide precise synthetic control over physicochemical properties and offer distinct advantages such as the conjugation of targeting ligands, endosomal escape, enhanced cellular uptake, and stimuli‐responsive drug release. These attributes position polymeric carriers as versatile platforms for optimizing cGAS‐STING agonist delivery. Polymersomes, in particular, exhibit a unique architecture, with hydrophobic membranes and aqueous cores, allowing for the simultaneous encapsulation of both hydrophilic and hydrophobic agents. For example, Wilson and colleagues engineered endosomolytic polymersomes with an aqueous core, specifically designed for efficient cGAMP delivery (Figure 4D).^[^ ^40^ ^]^ These vesicles consisted of amphiphilic diblock copolymers that exhibit pH‐responsive, endosomal membrane‐destabilizing properties, enabling the intracellular release of cGAMP and its escape from endosomes. The polymersome structure was engineered using PEG‐b‐[(2‐(diethylamino) ethyl methacrylate)‐co‐(butyl methacrylate)‐co‐(pyridyl disulfide ethyl methacrylate)] (PEG‐DBP), synthesized through reversible addition‐fragmentation transfer (RAFT) polymerization.^[^ ^40^ ^]^ The DEAMA‐co‐BMA segments exhibited membrane‐destabilizing activity, while thiol‐reactive pyridyl disulfide ethyl methacrylate (PDSMA) groups facilitated in situ crosslinking of polymer chains within the vesicle membrane. Under hydration conditions, these polymers self‐assembled into polymersomes, achieving an impressive 40% cGAMP encapsulation efficiency. Following intratumoral administration in a B16‐F10 murine melanoma model, these polymersomes reprogrammed the TME from an immunosuppressive to an inflamed and immunogenic state. Notably, in a dual‐tumor model, intratumoral injection into one tumor led to marked regression of both primary and distant tumors, demonstrating a strong abscopal effect.^[^ ^40^ ^]^

Despite these advancements, several challenges remain to be addressed before clinical translation. Key areas for further investigation include optimizing nanoparticle formulations to balance efficacy and safety, improving tumor‐targeting specificity, and understanding long‐term immunogenic effects. Moreover, the combination of these nanotherapeutics based on STING agonists with other therapeutic strategies could further enhance treatment outcomes. As research in this field continues to evolve, the development of next‐generation nanoplatforms with enhanced biocompatibility, precise immune modulation, and scalable manufacturing processes will be crucial for advancing STING agonist‐based immunotherapy towards clinical applications.

## 3 Emerging STING Agonists‐Based Nanotherapeutics in Combined Therapies

The activation of the STING signaling pathway has emerged as a potential therapeutic strategy in cancer immunotherapy owing to its role in initiating and amplifying endogenous antitumor immune responses.^[^ ^41^ ^]^ However, STING agonist‐based monotherapy has shown limited efficacy in preclinical and clinical settings, primarily due to high toxicity and frequent dosing requirements.^[^ ^42^ ^]^ Additionally, first‐generation CDN‐based STING agonists are mainly confined to intratumoral administration, hampering their applications in the treatment of metastatic, abscopal, deeply situated, or otherwise inaccessible malignancies.^[^ ^21^, ^28^ ^]^ Therefore, combining STING agonists with other current standard‐of‐care cancer therapies offers a synergistic strategy to enhance therapeutic outcomes and expand their clinical utility.^[^ ^43^ ^]^ This approach not only amplifies the immunostimulatory effects of STING activation but also helps overcome the immunosuppressive TME. Moreover, advancements in nanotechnology have further enabled the development of STING agonist‐based nanotherapeutics, improving delivery and efficacy across a broader range of tumor types.^[^ ^9^, ^44^ ^]^ This section reviews recent advances in combination therapies involving STING agonist‐based nanotherapeutics, with an emphasis on preclinical and clinical evidence supporting their synergistic effects. Key strategies include combining STING agonists with chemotherapy, radiotherapy, ICB, IDO inhibitors, cancer vaccines, ACT, phototherapy, SDT, and other modalities. Furthermore, the biological and mechanistic rationale for these combinations is discussed, providing valuable insights to guide the rational design of combination regimens that achieve durable and robust clinical outcomes.

### 3.1 STING Agonists‐Based Nanotherapeutics Combined with Chemotherapy

The therapeutic efficacy of chemotherapy has traditionally been attributed to its capacity to suppress tumor cell proliferation and induce cancer cell death by interfering with DNA replication, microtubule assembly, or cellular metabolism. However, emerging preclinical and clinical evidence suggests that certain chemotherapeutic agents also possess immunostimulatory properties. For instance, chemotherapeutic agents such as oxaliplatin, anthracyclines, and bortezomib can induce immunogenic cell death (ICD), an adaptive stress response characterized by the extracellular release of immune‐stimulating signals.^[^ ^45^ ^]^ These signals include the release of high mobility group box 1 (HMGB1), the secretion of ATP, and the surface exposure of endoplasmic reticulum chaperone (e.g., calreticulin, HSP70, and HSP90). Collectively, these damage‐associated molecular patterns (DMAPs) facilitate the uptake of TAAs by APCs, leading to antitumor immune activation.^[^ ^45^, ^46^ ^]^ While these findings support the potential of leveraging chemotherapy‐induced ICD in cancer immune combination therapies, the immunostimulatory effects of chemotherapy are often insufficient to elicit robust antitumor immunity due to the poor immunogenicity of tumor antigens, weak adjuvanticity of DAMPs, and the dominant presence of immunosuppressive TME.

Building on these findings, preclinical studies have revealed that DNA‐damaging chemotherapies can iatrogenically activate the STING pathway. Many classical chemotherapeutic agents, including paclitaxel,^[^ ^32c^ ^]^ doxorubicin (DOX),^[^ ^47^ ^]^ etoposide,^[^ ^48^ ^]^ platinum compounds,^[^ ^49^ ^]^ and camptothecins,^[^ ^50^ ^]^ can induce DNA damage, leading to the release of cytosolic dsDNA. Cytosolic dsDNA serves as a substrate for cGAS recognition, thereby activating the STING pathway and triggering type I interferon production. This activation also enhances MHC I antigen presentation, boosting T cell‐mediated tumor killing. These insights provide a mechanistic basis for the synergistic effects observed between DNA‐damaging chemotherapies and STING‐targeting immunotherapies. Consequently, combining DNA‐damaging chemotherapy with therapies that directly activate the STING pathway holds great potential for augmenting the therapeutic effectiveness by creating a pro‐inflammatory environment that fosters antitumor immunity.

The rationale for combining STING agonists with chemotherapy is threefold. First, STING agonist‐based immunotherapy can amplify and sustain the chemotherapy‐induced tumor immunity cycle. Second, chemotherapy‐mediated tumor debulking may allow STING agonists more time to act, reducing the likelihood of resistant clones emerging. Third, this combination could expand the therapeutic applicability of STING agonists across different cancer types and stages. Taken together, combining STING agonists with chemotherapy presents a rational strategy to overcome the limitations of both modalities, offering complementary mechanisms for enhanced antitumor efficacy. This combination has the potential to address critical obstacles in cancer treatment, thereby setting the stage for more effective combination regimens.

In recent years, increasing attention has been directed toward understanding the interplay between STING agonist‐based immunotherapy and conventional chemotherapy in generating potent antitumor immunity. Among the various chemotherapeutic agents, the combination of camptothecin derivatives with STING agonists has been extensively studied.^[^ ^50^, ^51^ ^]^ Notably, Cui et al. designed a supramolecular hydrogel co‐loaded with the STING agonist c‐di‐AMP and the chemotherapeutic agent camptothecin, representing a remarkable example of leveraging the synergy between these two therapeutic modalities.^[^ ^50b^ ^]^ In this work, a peptide‐drug conjugate, dicamptothecin‐iRGD, was synthesized by conjugating camptothecin to the tumor‐penetrating peptide iRGD via a reduction‐sensitive disulfanyl‐ethyl carbonate linker.^[^ ^50b^ ^]^ The drug‐peptide conjugate could self‐assemble into nanotubes with positively charged surfaces, facilitating electrostatic complexation of the negatively charged c‐di‐AMP. Upon intratumoral administration, the hydrogel formed instantaneously, acting as a sustained drug reservoir for localized release of both camptothecin and c‐di‐AMP over at least two weeks. The therapeutic potential of this system was demonstrated across multiple tumor models, including 4T1 breast cancer, GL‐261 glioma, and CT26 colon cancer. A single dose of the hydrogel significantly suppressed tumor growth and extended survival relative to control groups: a soluble mixture of camptothecin and c‐di‐AMP, a hydrogel without camptothecin, and a hydrogel lacking c‐di‐AMP.^[^ ^50b^ ^]^ These findings underscore the promise of combining STING agonists with camptothecin‐based chemotherapies to achieve durable antitumor immunity and improved therapeutic outcomes.

Building on the success of locally administered delivery systems, subsequent efforts have shifted towards developing systemic delivery strategies for combination therapy. For instance, Liang et al. designed a redox‐responsive polymeric prodrug nanosystem for the codelivery of STING agonist DMXAA and the DNA‐targeting chemotherapeutic agent SN38 (7‐ethyl‐10‐hydroxycamptothecin).^[^ ^50c^ ^]^ This system was constructed utilizing a triblock copolymer synthesized via RAFT polymerization. The copolymer consisted of a hydrophilic PEG block, a second hydrophobic block containing a redox‐responsive SN38 prodrug monomer, and a third block of diethylaminoethyl methacrylate (DEAMA, designed to electrostatically interact with the carboxyl group of DMXAA.^[^ ^50c^ ^]^ The resulting amphiphilic polymer self‐assembled into nanoparticles (PS3D1@DMXAA) with a particle size of ∼30 nm and a DMXAA loading efficiency of approximately 14%. When administered intravenously, PS3D1@DMXAA exhibited superior efficacy in suppressing tumor progression and metastasis in murine models of melanoma and breast cancer. Strikingly, its antitumor activity was significantly greater than the combo of free DMXAA with SN38‐loaded nanoparticles (PS3D1) or free DMXAA alone. These improved therapeutic benefits were ascribed to the synergistic interaction between SN38 and DMXAA, which converted the TME from immunosuppressive to immunostimulatory. This transformation facilitated the activation of APCs and increased CD8+ T cell infiltration into tumors, thereby potentiating antitumor immunity.^[^ ^50c^ ^]^

**Figure 5:**
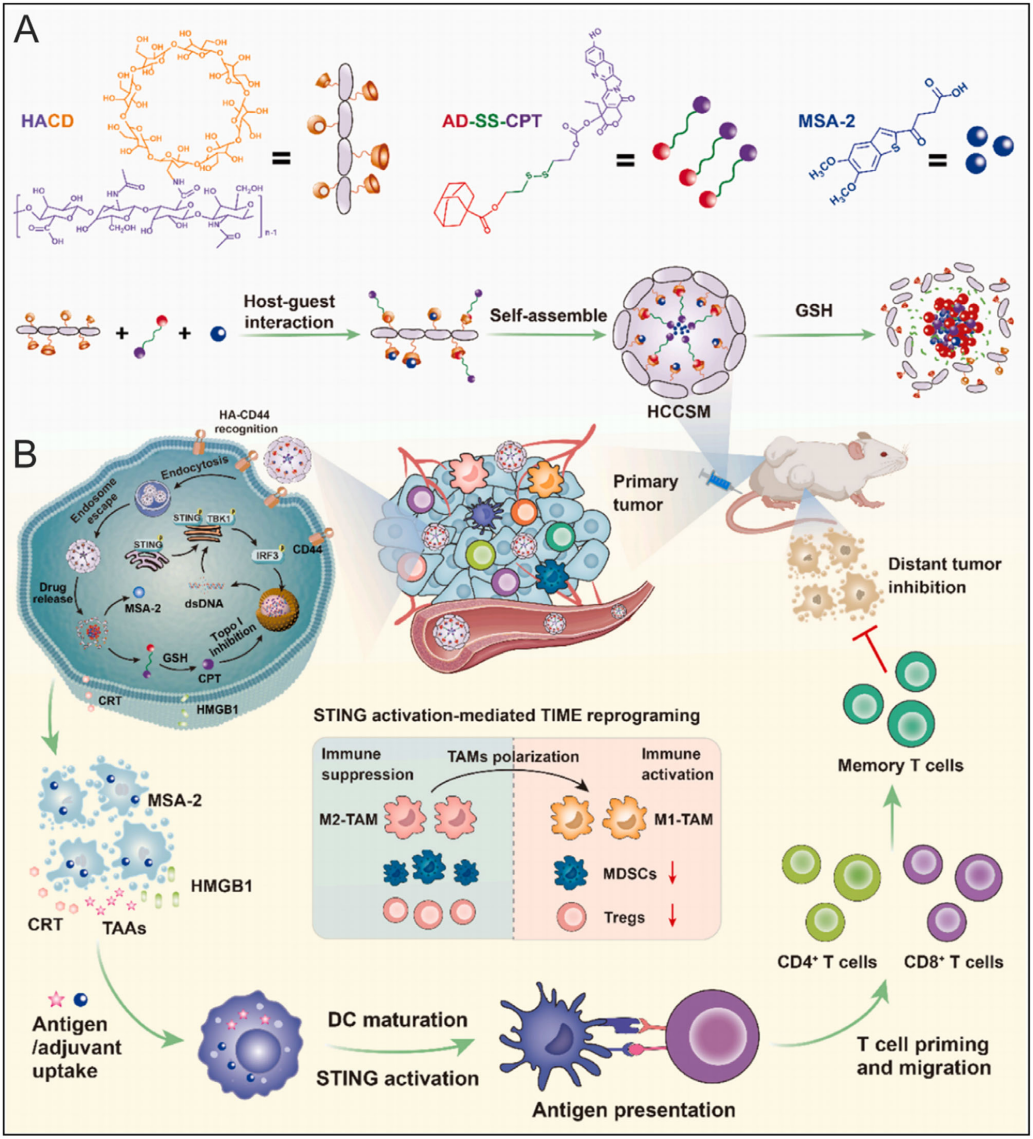
Schematic illustration of the STING agonist‐integrated supramolecular nanovector for in situ vaccination immunotherapy of colorectal cancer. (A) Schematic diagram of the nanovector preparation by supramolecular self‐assembly of HA‐CD with CPT prodrug and STING agonist MSA‐2 via the host‐guest interaction. (B) Schematic diagram of the nanovector‐elicited antitumor immunity via ICD induction and cGAS‐STING activation to inhibit primary tumor growth and distant metastasis. Reproduced with permission. ^[^ ^51^ ^]^ Copyright 2024, Elsevier.

While polymeric nanosystems offer significant advantages for systemic delivery, host‐guest chemistry has emerged as an alternative strategy to enhance drug solubility, stability, and targeting specificity. Leveraging this approach, Yu et al. synthesized an adamantane ‐conjugated camptothecin (CPT) heterodimer through a reduction‐responsive disulfide bond, named AD‐SS‐CPT (**Figure** **5**). The yielded prodrug, combined with the STING agonist MSA‐2, was assembled with cyclodextrin‐grafted hyaluronic acid (HA‐CD) to form a supramolecular prodrug nanovector, designated HCCSM, with an average diameter of appropriately ≈104.3 nm.^[^ ^51^ ^]^ Intravenous administration of HCCSM nanovectors in a subcutaneous CT26 colorectal tumor model significantly delayed the tumor progression of both primary and distant tumors compared to controls treated with a soluble formulation of CPT+MSA‐2 or CCSM nanovectors lacking HA modification. Mechanistic studies revealed that the synergistic antitumor effects of HCCSM were associated with enhanced STING pathway activation, as evidenced by elevated expression of phosphorylated TBK1 and IRF3.^[^ ^51^ ^]^ This study provides compelling evidence for the potential of combining chemotherapy with STING agonists to elicit robust immune activation and establish in situ vaccination effects for improved therapeutic outcomes.

In addition to CPT‐based drugs, STING agonists have also been investigated in combination with other chemotherapeutic agents, including anthracyclines,^[^ ^52^ ^]^ platin‐based drugs,^[^ ^53^ ^]^ and nucleoside metabolic inhibitors.^[^ ^54^ ^]^ For example, Hou et al. reported a multifunctional drug delivery system comprising DOX and amorphous porous manganese phosphate (APMP) for combining chemotherapy with STING agonist‐based immunotherapy.^[^ ^52a^ ^]^ This nanoplatform was fabricated via encapsulating DOX within APMP NPs, followed by a phospholipid (PL) coating to enhance stability during blood circulation and phospholipase‐mediated biodegradation at the tumor sites. Following intravenous administration, DOX released at the tumor site induced DNA damage and activated the cGAS pathway, while Mn^2+^ synergistically potentiated the cGAS‐STING signaling cascade.^[^ ^52a^ ^]^ As a result, treatment with PL/APMP‐DOX NPs in a murine breast cancer model significantly enhanced the tumor infiltration of DCs, activated tumor‐infiltrating CD8^+^ T cells and NK cells, and demonstrated robust chemo‐immune synergy through DOX‐induced STING activation and Mn^2+^‐mediated STING signaling potentiation.^[^ ^52a^ ^]^

Similarly, Zhou et al. engineered a multifaceted inorganic nanoplatform, Mn_3_O_4_@Au‐dsDNA/DOX, utilizing dsDNA‐Au conjugates and DOX to co‐assemble onto Mn_3_O_4_ nanoflowers.^[^ ^52b^ ^]^ The poly(dT) single‐stranded DNA was first conjugated onto Au nanoparticles (AuNPs) via Au‐S bonds, annealed with complementary strands to form Au‐dsDNA, and then co‐assembled with DOX onto Mn_3_O_4_ nanoflowers. This nanoplatform effectively delivered dsDNA, Mn^2+^, and DOX to tumors following intravenous injection. Within tumor cells, the nanoparticles decomposed to release dsDNA, Mn^2+^, and DOX, thereby enhancing STING pathway activation and triggering a strong antitumor immune response.^[^ ^52b^ ^]^ More importantly, the NPs effectively decreased tumor growth in both 4T1 and B16‐F10 models and prolonged survival rates, demonstrating significant potential for synergy between chemotherapy and STING agonist‐based immunotherapy.^[^ ^52b^ ^]^

Apart from the aforementioned inorganic nanoparticles, research has been dedicated to developing liposomal formulations as an alternative strategy for the co‐delivery of STING agonists and anthracycline drugs.^[^ ^52c,d^ ^]^ Li et al. constructed a library of cationic liposomal formulations consisting of varying proportions of cationic lipid, 1,2‐dioleoyl‐3‐trimethylammonium‐propane (DOTAP) along with 1,2‐distearoylsn‐glycero‐3‐phosphocholine (DSPC), 1,2‐dioleoyl‐sn‐glycero‐3 phosphoethanolamine (DOPE), 1,2‐dimyristoyl‐rac‐glycerolmethoxy (poly (ethylene glycol)) (DMG‐PEG2000) and cholesterol. These liposomes, termed DGL, were co‐loaded with the chemotherapeutic drug DOX and the STING agonist cGAMP for combined chemo‐immunotherapy.^[^ ^52c^ ^]^ Systematic screening revealed that formulations containing 8% DOTAP optimized in lysosomal escape and tumor distribution of cGAMP and DOX, resulting in an 86% tumor inhibition rate and prolonged survival in B16‐F10 tumor‐bearing mice. Mechanistic studies highlighted the immune‐related basis of the combinatorial antitumor efficacy, demonstrating increased ICD, enhanced STING pathway activation, and greater infiltration of CD8+ effector T cells in the tumors of mice treated with DGL.^[^ ^52c^ ^]^

Building upon liposomal platforms designed for the co‐delivery of chemotherapeutic drugs and STING agonists, recent efforts have focused on tackling the specific challenges of postsurgical tumor recurrence. Cui et al. developed a neutrophil‐hitchhiking liposomal system for targeted chemo‐immunotherapy at postsurgical tumor sites.^[^ ^52d^ ^]^ This liposomal platform was strategically functionalized with anti‐Ly6G antibodies to specifically bind neutrophils, enabling their transport to postsurgical inflamed tumor sites. Upon intravenous administration, the liposomes efficiently attached to circulating neutrophils, which subsequently migrated to the tumor resection site. The released DOX eradicated residual lesions and induced ICD, activating tumor‐specific immunity. Concurrently, SR‐717, a non‐nucleotide STING agonist, robustly activated the STING pathway, providing an immunostimulatory milieu that promoted DC maturation and T cell priming. As a result, the liposomes co‐loaded with DOX and SR‐717 elicited strong antitumor immune responses in melanoma models, effectively suppressing tumor recurrence and metastatic growth.^[^ ^52d^ ^]^ This study not only demonstrated the potential synergy between STING activation and chemotherapy but also highlighted the innovative use of neutrophil‐mediated delivery for enhancing therapeutic targeting.

While liposomal carriers have achieved notable translational success in drug co‐delivery systems, exemplified by FDA‐approved formulations such as CPX‐351, polymeric carriers offer distinct advantages. These include precise synthetic control over physicochemical properties and the capacity for multifunctional integration, making polymers also promising for the co‐delivery of therapeutic agents. For instance, Lee et al. developed a polypeptide‐based nanocarrier to co‐deliver DOX and the diABZI STING agonist 3 (dSA3),^[^ ^52e^ ^]^ achieving APC‐specific delivery and tumor phagocytosis‐driven STING pathway activation. The resulting nanoparticles (DSNs) demonstrated remarkable efficacy in inhibiting tumor growth and elicited robust innate and adaptive immune responses within the TME and spleen in TC1 and MC38 tumor models. Furthermore, functionalization with the targeting ligand cRGD enhanced their therapeutic efficacy, achieving pathological remission in primary tumors and immune‐mediated rejection of rechallenged tumors.^[^ ^52e^ ^]^ These findings highlight the significant potential of polymeric nanocarriers for co‐delivery applications and demonstrate the synergy between STING agonists and chemotherapy in combination cancer therapy.

An example that highlights the synergistic potential of platin‐based drugs and STING agonists in combined cancer immunotherapy was reported by Fan et al., who developed Mn‐doped mesoporous silica nanoparticles (MM) featuring an acidity/redox‐responsive metal‐organic framework (MOF, ZIF8) as a functional gate.^[^ ^53^ ^]^ The nanoparticles were co‐loaded with cisplatin, Mn^2+^, and the STING agonist SR‐717 to enable cascade activation of the cGAS‐STING pathway. Specifically, cisplatin was encapsulated within the mesopores of the MM, which was then capped with the MOF loaded with SR‐717 to create PMM@MOF@SR‐717 (PMMR), effectively preventing drug leakage. This structure was further cloaked with an erythrocyte membrane and modified with HA, yielding the final formulation PMMR@eM‐HA (PMMR@MH). In 4T1‐luc breast tumor models, PMMR@MH achieved complete tumor inhibition, significantly outperforming nanoparticles loaded with cisplatin (PMM@MH), SR‐717 (MMR@MH), or Mn^2+^ (MM) alone.^[^ ^53^ ^]^ These results underscore the capability of PMMR@MH to integrate chemotherapy, metallotherapy, and immunotherapy into a synergistic strategy, thereby generating potent antitumor effects.

Overall, these findings provide compelling evidence that STING agonists, when co‐encapsulated with DNA‐damaging chemotherapeutic agents in rationally designed delivery systems, can synergistically enhance antitumor immunity, offering a promising strategy for combined cancer therapy.

### 3.2 STING Agonists‐Based Nanotherapeutics Combined with Radiotherapy

Radiotherapy is an effective therapeutic modality for treating unresected tumor lesions and preventing locoregional relapse after surgery.^[^ ^55^ ^]^ Traditionally, whole‐body radiotherapy often led to pronounced declines in blood cell counts due to systemic bone marrow exposure to large radiation fields, thus reinforcing the perception that radiation is generally immunosuppressive.^[^ ^56^ ^]^ However, advancements in highly focused radiation techniques, such as proton therapy and stereotactic body radiotherapy, alongside the development of nanotechnology, have enabled precise delivery of radiopharmaceuticals while substantially reducing the radiation fields. These advancements minimize the radiation damage to neighboring healthy tissues and allow higher radiation doses to be utilized to control tumor growth with reduced toxicity.^[^ ^55^ ^]^ The above fundamental change necessitates a reinterpretation of the immunological effects of modern radiotherapy.

Although immunosuppression caused by localized radiation still exists, a growing body of evidence shows that radiotherapy can also bring about multiple immunostimulatory functions beyond its DNA‐damaging properties. These include the release of TAAs and DAMPs, remodeling of the immunosuppressive TME, induction of ICD, and even abscopal responses in non‐irradiated lesions. The immunological effects of radiation have been comprehensively reviewed elsewhere.^[^ ^55^, ^57^ ^]^ These findings have defined the rationale for combining radiotherapy with immunotherapeutics.

A key mechanism linking radiotherapy to immune activation involves the STING pathway. Radiotherapy kills cancer cells via inducing non­repairable DNA damage,^[^ ^55^ ^]^ resulting in the accumulation of cytosolic dsDNA in tumor cells, which can activate the STING signaling pathway through cGAS recognition. This initiates downstream signaling, including proinflammatory cytokines and type I interferons production, thereby promoting both innate and adaptive antitumor immunity.^[^ ^8^, ^12^, ^58^ ^]^ However, this radiotherapy‐induced iatrogenic STING activation is transient and consequently insufficient to produce durable antitumor effects.^[^ ^59^ ^]^ Moreover, radiotherapy‐induced tumor immunogenicity is thought to be suppressed by DNA exonuclease TREX1, which degrades oxidized dsDNA at high radiation doses and attenuates STING activation.^[^ ^60^ ^]^ Although radiotherapy can activate the STING pathway and induce ICD, it remains largely ineffective as a monotherapy for generating durable anticancer immunity.^[^ ^61^ ^]^ In addition, radiotherapy promotes TGF‐β production by local tumor cells, which facilitates the recruitment of regulatory T cells (Tregs) and contributes to the establishment of an immunosuppressive TME. Consequently, combining low‐dose radiotherapy with STING agonists may robustly enhance systemic immune responses and provide durable therapeutic benefits.

Currently, integrating radiotherapy with STING agonist‐based nanotherapeutics represents a promising approach to maximize therapeutic efficacy in cancer therapy. Thereinto, a notable example of synergistic approach is demonstrated in the synergistic treatments of metastatic lung cancer.^[^ ^62^ ^]^ Liu et al. developed an innovative inhalable liposomal cGAMP formulation specifically designed for pulmonary delivery and evaluated its anti‐metastasis effects in combination with radiotherapy.^[^ ^62^ ^]^ To target pulmonary APCs, the liposomes were coated with phosphatidylserine, which is recognized by macrophages and DCs as a marker on apoptotic cells. Additionally, cGAMP was encapsulated in the liposomal core using calcium phosphate to ensure effective intracellular release.^[^ ^62^ ^]^ This design facilitated the aerosolized liposomal cGAMP to enhance IFN‐I production and achieve significant synergistic effects with radiotherapy, surpassing the efficacy of monotherapy in two lung metastasis mouse models of 4T1 breast cancer and B16‐OVA melanoma.^[^ ^62^ ^]^

Similarly, other CDN‐based STING agonists have demonstrated significant therapeutic potential in this combinatorial approach. Zheng et al. developed reduction‐responsive, biodegradable chimeric polymersomes (termed CPs‐CDN) capable of efficiently encapsulating and delivering the first‐generation synthetic CDN, ADU‐S100.^[^ ^59b^ ^]^ This nanoparticle platform demonstrated a stable and high drug‐loading capacity, addressing one of the key challenges in CDN delivery. When combined with low‐dose fractionated radiotherapy, CPs‐CDN exhibited strong synergistic antitumor activity against B16F10 melanoma in vivo, leading to robust tumor regression. Furthermore, this combination induced long‐term immune memory, effectively protecting mice from tumor rechallenge.^[^ ^59b^ ^]^

Building on these principles, further advancements have focused on combining STING agonists with multifunctional radiosensitizing platforms to maximize therapeutic outcomes.^[^ ^63^ ^]^ Lin et al. developed an innovative 2D nanoplatform, cGAMP/MOL, designed to synergistically combine radiotherapy and STING agonist‐based immunotherapy.^[^ ^63^, ^64^ ^]^ This platform utilizes a nanoscale metal‐organic layer (MOL) constructed from Hf_12_ secondary building units and DBB‐Ir photosensitizer ligands, which serve not only as radiosensitizers but also as ideal scaffolds for cGAMP conjugation, enabling precise functionalization and enhancing therapeutic efficacy.^[^ ^63^ ^]^ The resulting cGAMP/MOL significantly enhanced radiosensitization, promoted tumor cell death, and induced ICD, while simultaneously enabling sustained cGAMP retention within the TME for prolonged STING pathway activation. Compared to free cGAMP, cGAMP/MOL markedly amplified STING pathway activation and demonstrated superior tumor regression following X‐ray irradiation. In two murine colon cancer models, the combination of radiotherapy and STING activation achieved robust local tumor control and effectively enhanced T‐cell infiltration, while potentially reducing immunosuppressive cell populations.^[^ ^63^ ^]^ Another innovative system was developed by Zhang et al., who integrated the catalytic radiosensitizer DMPtNPS with the STING agonist cGAMP to address hypoxia‐induced radioresistance and enhance radioimmunotherapy efficacy.^[^ ^64^ ^]^ DMPtNPS efficiently facilitated X‐ray energy transfer to produce reactive oxygen species (ROS), while alleviating tumor hypoxia to increase radiosensitivity. To counteract the immunosuppressive effects of DMPtNPS and radiotherapy on the TME, cGAMP was incorporated into DMPtNPS, forming DMPtNPS@cGAMP. This combined treatment of radiotherapy and DMPtNPS@cGAMP resulted in durable complete tumor regression at the primary site and a strong abscopal effect at distant sites in a rectal cancer model.^[^ ^64^ ^]^ These studies collectively highlight the potential of integrating STING agonists with radiosensitizers to redefine cancer treatment paradigms, offering a compelling strategy that redefines the benchmark for synergistic cancer treatment strategies, paving the way for novel radiotherapy‐immunotherapy combinations.

In addition to the aforementioned nanoformulations of CDN‐like STING agonists, researchers have also explored metal‐coordinated nanoplatforms to further boost radiotherapy efficacy.^[^ ^65^ ^]^ An exemplary study showing that Mn^2+^ can synergize well with radiotherapy was published by Wang et al., who designed a nanoplatform termed TMA‐NPs, composed of c‐di‐AMP, Mn ion, and tannic acid.^[^ ^65a^ ^]^ Phenolic hydroxyl groups in tannic acid enable both intermolecular hydrogen bonding with drug molecules and robust metal‐phenolic coordination, providing a plethora of anchoring sites for Mn ions and c‐di‐AMP. This unique design allowed TMA‐NPs to enhance X‐ray irradiation‐induced STING pathway activation in murine breast cancer models. As a result, TMA‐NPs considerably improved the therapeutic efficacy of radiotherapy, inhibiting both primary large tumors and distant tumor growth.^[^ ^65a^ ^]^ Mechanistically, the elevated levels of c‐di‐AMP triggered robust STING signaling cascades, promoting DC maturation and T lymphocyte activation, ultimately enhancing local and systemic antitumor immune responses.^[^ ^65a^ ^]^

**Figure 6:**
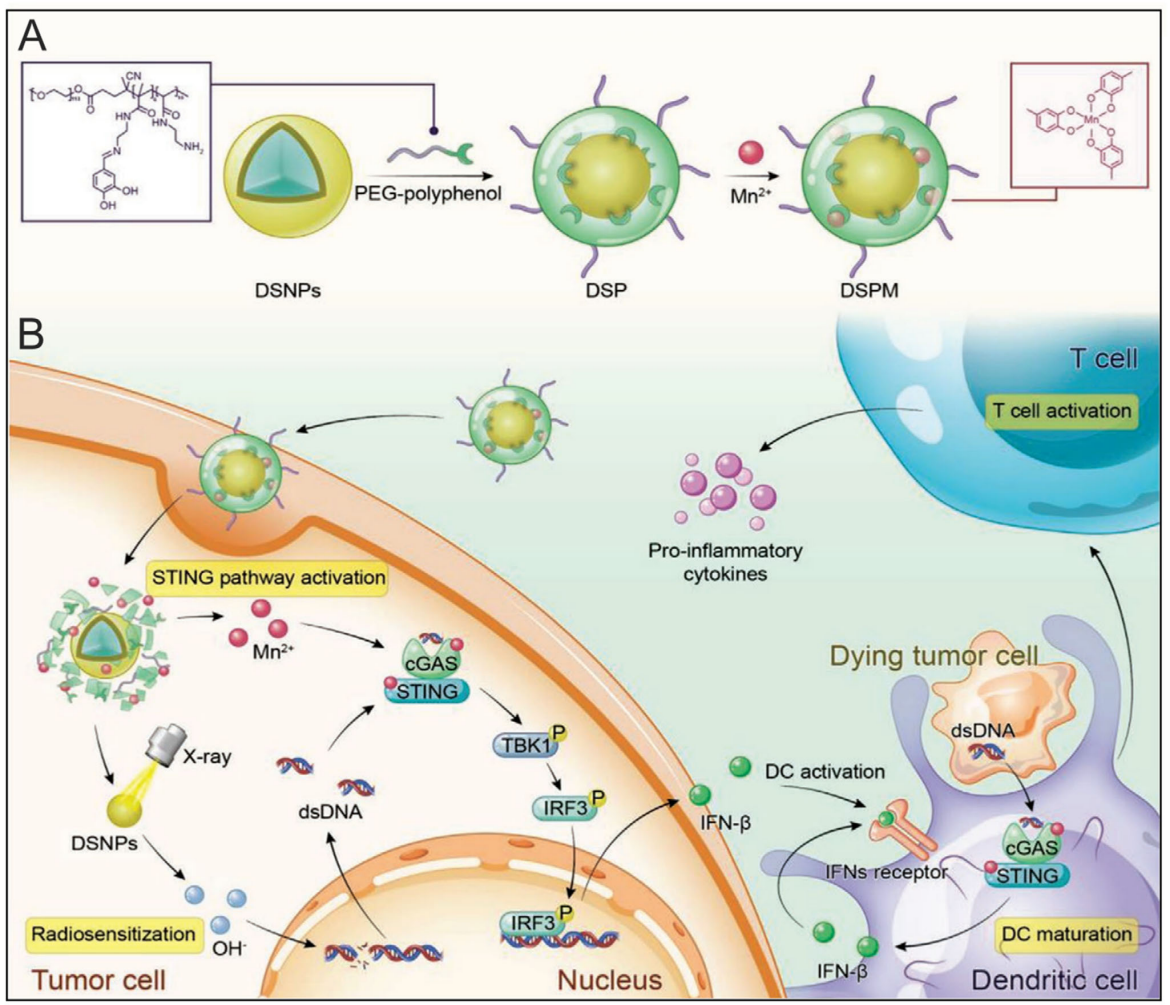
Schematic illustration of the preparation and biological function of DSPM. (A) Schematic diagram of the preparation routes of DSPM. (B) Schematic illustration of DSPM‐mediated radiosensitization and STING pathway‐dependent antitumor immunity. After cellular uptake of DSPM, pH stimuli‐responsive PEG‐polyphenol disassembled and released DSNPs and Mn^2+^. Upon X‐ray irradiation, DSNPs induced cancer cells death and resulted in the generation of cytosolic dsDNA. Meanwhile, the released Mn^2+^ sensitized cGAS to cytosolic dsDNA for STING‐mediated IFN‐β production in both cancer cells and DCs, resulting in DC maturation and robust antitumor immunity. Reproduced with permission.^[^ ^65b^ ^]^ Copyright 2022, John Wiley & Sons.

Additionally, Yan et al. leveraged the coordination between metal ions and phenolic hydroxyl groups to develop a metal‐phenolic network (DSPM) for the dual‐delivery of a Mn^2+^ and lanthanide‐doped radiosensitizer (NaGdF_4_:Nd@NaLuF_4_) (**Figure** **6**).^[^ ^65b^ ^]^ To accomplish this, hydrophobic core‐shell NaGdF_4_:Nd@NaLuF_4_ nanoparticles were synthesized via coprecipitation and coated with an amphiphilic PEG‐polyphenol polymer, which facilitated Mn^2+^ coordination and enabled the formation of the final formulation DSPM. In the acidic TME, DSPM disassembled due to imine linkage cleavage in PEG‐polyphenol, allowing the release of both Mn^2+^ and NaGdF_4_:Nd@NaLuF_4_. Upon X‐ray irradiation, the released NaGdF_4_:Nd@NaLuF_4_ sensitized tumor cells, promoting the release of cytosolic dsDNA. Simultaneously, Mn^2+^ potentiated the dsDNA recognition by cGAS, amplifying STING pathway activation.^[^ ^65b^ ^]^ Following intravenous administration, the combination of NaGdF_4_:Nd@NaLuF_4_ and Mn^2+^ under X‐ray irradiation demonstrated robust synergistic effects in a 4T1 tumor model. The DSPM significantly suppressed both primary and distant tumor growth while enhancing antitumor immune responses when combined with radiotherapy.^[^ ^65b^ ^]^ Although alternative timing or sequencing regimens were not explored, this study demonstrated that co‐delivery of a STING agonist and a radiosensitizer can improve therapeutic efficacy. Furthermore, it motivates the design of nano‐DDSs leveraging Mn ion coordination to boost synergistic effects between radiotherapy and STING activation.

Taken together, these data collectively emphasize the potential of integrating STING activation with radiotherapy to enable beneficial antitumor effects and redefine cancer treatment paradigms. Future research should focus on optimizing these strategies and exploring their clinical translation to maximize therapeutic outcomes.

### 3.3 STING Agonists‐Based Nanotherapeutics Combined with Immunotherapy

Cancer immunotherapies, such as ICB and chimeric antigen receptor (CAR) T‐cell therapy, have revolutionized oncology and are now established as a cornerstone of cancer therapy alongside surgery, chemotherapy, and radiotherapy. Despite the regulatory approval of over 20 immunotherapeutic products (multiple therapies) and numerous in‐depth ongoing clinical trials, significant challenges still hinder the broad clinical application of cancer immunotherapies.

One major obstacle is that many patients either fail to respond to immune‐based therapeutic strategies or experience only limited clinical benefits, with some even displaying intrinsic resistance.^[^ ^66^ ^]^ Clinical data show that only a certain subset of patients, typically with immunogenic “hot tumors”, demonstrate durable clinical responses to ICB therapy, with response rates of 10%–30%, depending on the cancer type. In contrast, patients with nonimmunogenic “cold tumors” generally exhibit poor responses.^[^ ^67^ ^]^ Therefore, to maximize the potential of cancer immunotherapy, additional strategies are urgently needed to convert “cold tumors” into “hot tumors”.

A substantial amount of preclinical and clinical evidence suggests that the efficacy of immunotherapies such as ICB typically relies on pre‐existing proinflammatory antitumor responses. Therapeutic approaches that trigger *de novo* innate immune activation and enhance immune priming in nonimmunogenic tumors could significantly expand the types of cancers effectively targeted by immunotherapies. Furthermore, strategies aimed at promoting immune priming and enhancing T‐cell infiltration are poised to complement current immunotherapeutics. In this context, substantial efforts are focused on introducing immune stimulators, such as STING agonists, to boost response rates. Notably, several clinical trials are currently investigating the combination of STING agonists with ICBs, and promising clinical results are driving the integration of STING‐based therapies with other immune‐based cancer treatment strategies.

Despite numerous ongoing preclinical and clinical studies exploring the impact of STING agonists and other immunotherapies on effective antitumor immunity, a comprehensive understanding of the mechanistic rationale underlying these combinatorial strategies remains elusive. This section examines emerging evidence on the complex interplay between STING agonists and immunotherapies, including ICBs, IDO inhibitors, cancer vaccines, and ACT. Representative examples are also discussed to provide insight into the underlying scientific rationale behind these integrated approaches.

#### 3.3.1 STING Agonists‐Based Nanotherapeutics Combined with ICBs

Targeting key immune checkpoint molecules, such as programmed cell death‐1 (PD‐1), programmed cell death 1 ligand 1 (PD‐L1), and cytotoxic T lymphocyte antigen‐4 (CTLA‐4), has achieved remarkable breakthroughs in multiple solid tumors by blocking immunoinhibitory signals and facilitating the generation of potent antitumor responses. However, despite these successes, several key challenges remain for cancer ICB therapies. As aforementioned, many patients do not respond to single‐agent or dual‐agent ICB therapies. Factors contributing to ICB resistance are being intensely investigated and may include limited tumor‐infiltrating T cells, dysregulated immune checkpoint signaling in tumor and T cells, and adaptive immune resistance mechanisms. Additionally, immunosuppressive TME necessitates the development of innovative, integrated approaches to improve treatment outcomes.

Although dual ICBs have demonstrated improved therapeutic efficacy by amplifying antitumor immune responses, they concurrently exacerbate immune‐related adverse effects due to the disruption of immune homeostasis.^[^ ^68^ ^]^ These limitations may be effectively addressed by combining ICBs with immune‐stimulating agents, such as STING agonists, which can synergistically activate antitumor immunity. Preclinical and early‐phase clinical studies have reported substantial increases in antitumor immune responses when ICB is combined with STING agonists. This section discusses emerging preclinical evidence supporting the potential of STING agonists to overcome ICB resistance, enhance therapeutic efficacy, and elucidate the mechanistic rationale underlying their combinatorial synergy. Representative studies will also be highlighted to provide a comprehensive understanding of this promising therapeutic strategy.

Notably, existing evidence suggested that STING agonist‐based monotherapy modulates the expression of key adaptive immune checkpoint molecules, including PD1 and CTLA‐4 on T cells as well as PD‐L1 on tumor cells and immune cells like macrophages and DCs within the TME. This modulation is not overly surprising, as STING agonists are known to potentiate antitumor immunity by inducing proinflammatory cytokine production and type I interferon responses. Furthermore, IFN‐γ secreted by T cells can upregulate PD‐L1 expression on cancer cells,^[^ ^69^ ^]^ serving as a key negative mechanism to prevent autoimmune responses against tumor cells. Based on this, an intriguing possibility is that STING agonists could sensitize tumors to PD1/PD‐L1 blockade therapies by increasing PD‐L1 abundance on tumor cells, thereby improving the binding efficiency of PD‐1/PD‐L1 antibodies. Accordingly, combining PD‐1/PD‐L1 antibodies with STING agonists is a promising strategy to augment antitumor immune responses, particularly in settings where ICB treatment alone is ineffective. Conversely, PD‐L1 blockade may further amplify the antitumor effects of STING agonists, allowing for dose reductions of STING agonists to mitigate dose‐limiting toxicities.^[^ ^70^ ^]^ Mechanistically, the synergy between STING agonists and ICB therapy arises from their complementary activation of both innate and adaptive immune responses, thereby sensitizing tumors to ICB treatment and overcoming adaptive immune resistance.

On the basis of these findings, there is increasing focus on developing effective combination therapies involving ICBs and STING agonists, as evidenced by the increasing number of clinical trials targeting PD1, PD‐L1, or CTLA‐4. While these combinations have demonstrated improved clinical responses, challenges such as adverse immune‐related events and the inherent limitations of STING agonists remain to be addressed. Most recently, the integration of STING agonist‐based nanotherapeutics with ICB has emerged as a promising approach to maximize the therapeutic potential of immunotherapy.

Preclinical studies have shown that combining PD‐1 antibodies with STING agonists improves antitumor immune responses by overcoming the adaptive immune resistance induced by STING agonists or cytokine monotherapies. In a study by Cui and colleagues, a bioresponsive supramolecular hydrogel system was constructed for localized delivery of three immunomodulators and their combinations, including a STING agonist (CDA), aPD1 antibody, and an IL15 cytokine.^[^ ^71^ ^]^ The system was constructed using the amphiphilic DOCA‐PLGLAG‐iRGD, which conjugates hydrophilic iRGD peptide and hydrophobic deoxycholic acid (DOCA) to the C‐ and N‐termini of the MMP‐2 responsive PLGLAG peptide, respectively. This amphiphile spontaneously assembled into supramolecular filaments (SF) and formed an in situ hydrogel upon intratumoral injection, serving as a scaffold‐depot for sustained, MMP‐2‐responsive release of immunomodulators over time.^[^ ^71^ ^]^ In a subcutaneous GL‐261 glioma model, single‐agent immunotherapies (IL15‐SF or CDA‐SF) strongly inhibited tumor growth but induced adaptive immune resistance by upregulating PD‐1 and PD‐L1 expression.^[^ ^71^ ^]^ Importantly, combination therapies, such as aPD1/CDA‐SF and aPD1/IL15‐SF, substantially augmented T‐cell infiltration and alleviated immunosuppression compared to single‐agent treatments (CDA‐SF or IL15‐SF). These combinations effectively overcame immune resistance caused by CDA‐SF and improved long‐term survival in the GL‐261 glioma model while providing protection against tumor rechallenge.^[^ ^71^ ^]^ This study demonstrates the promise of supramolecular hydrogels for rational combination immunotherapy, offering a promising strategy to overcome treatment resistance and achieve durable antitumor immunity.

STING agonists combined with ICBs represent a promising strategy to enhance tumor sensitivity and improve the effectiveness of ICB therapy. For example, Zhang et al. leveraged the vascular extravasation and tissue‐penetration properties of neutrophils to develop a tumor‐penetrating, neutrophil‐based cytopharmaceutical system (NEs@STING‐Mal‐NP) to facilitate the delivery of STING agonists and reinvigorate the immunosuppressive microenvironment of triple‐negative breast cancer (TNBC).^[^ ^72^ ^]^ In this approach, hyaluronic acid‐maleimide (HAMal)‐modified cationic liposomal STING agonists were conjugated to neutrophils as a backpack through the reaction between maleimide and free thiols on the neutrophil surface.^[^ ^72^ ^]^ Following intravenous administration, NEs@STING‐Mal‐NP transmigrated across the tumor vascular endothelium and extravasated into the tumor site. Once in the TME, liposomal STING agonists were released through hyaluronidase‐mediated cleavage, augmenting the cellular internalization of STING agonists by tumor cells.^[^ ^72^ ^]^ As a result, NEs@STING‐Mal‐NP treatment significantly increased the infiltration of macrophages, DCs, NEs, and CD8^+^ T cells, reinvigorating the immunosuppressive microenvironment of TNBC.^[^ ^72^ ^]^ Remarkably, the combination of NEs@STING‐Mal‐NP with an anti‐PD‐1 antibody achieved a tumor inhibition rate of approximately 90.6%, a dramatic improvement over αPD‐1 monotherapy. Moreover, complete tumor regression was observed in 33% of mice with established orthotopic TNBC.^[^ ^72^ ^]^ These results underscore the potential of a neutrophil‐based delivery system as a versatile platform to synergize with ICBs, offering a powerful approach to overcome treatment resistance in aggressive cancers like TNBC.

Similar to anti‐PD‐1 antibodies, several preclinical studies have demonstrated the synergistic effects of STING agonists and anti‐PD‐L1 immunotherapy.^[^ ^70^, ^73^ ^]^ For instance, Doshi et al. developed a CD103^+^ DC‐targeted cationic liposome encapsulating the STING agonist ADU‐S100 using Clec9a targeting peptide.^[^ ^70^ ^]^ Administration of low doses of Clec9a‐targeted liposomes (SA‐TL) led to systemic antitumor immunity and sensitized tumors to ICB. Importantly, the combination of SA‐TL with anti‐PD‐L1 antibody resulted in an obvious increase in M1‐like macrophages (CD11b^+^F4/80^+^MHCII^+^) as well as CD8^+^IFNγ^+^ T cells, leading to superior antitumor efficacy compared with PD‐L1 antibody or SA‐TL monotherapy in subcutaneous MC38 and B16F10 tumor models.^[^ ^70^ ^]^ This study underscores the promise of DC‐targeted STING agonists to synergize with PD‐L1 blockade, providing a novel strategy to overcome immune resistance and improve antitumor immune responses.

In line with these findings, recent efforts have focused on integrated co‐delivery strategies that simultaneously incorporate both STING agonists and PD‐L1 antibodies into a single nanoplatform to achieve immunotherapeutic synergy. For instance, Xu et al. developed a multifunctional nanovesicle (GP@DMX NV) by encapsulating the STING agonist DMXAA into plant‐derived nanovesicles from the roots of Glycyrrhiza uralensis Fisch, followed by surface modification with PD‐L1 antibodies via DSPE‐PEG conjugation.^[^ ^73a^ ^]^ Intratumoral administration of GP@DMX NV in the B16‐F10 melanoma model significantly remodeled the TME, promoting DC maturation and CD8+ T cell infiltration. This co‐delivery system elicited a robust synergistic antitumor response and effectively sensitized tumors to ICB.^[^ ^73a^ ^]^ This study highlights the promise of multifunctional nanovesicles in coordinating innate and adaptive immunity, offering a compelling strategy to potentiate combinatorial cancer immunotherapy.

**Figure 7:**
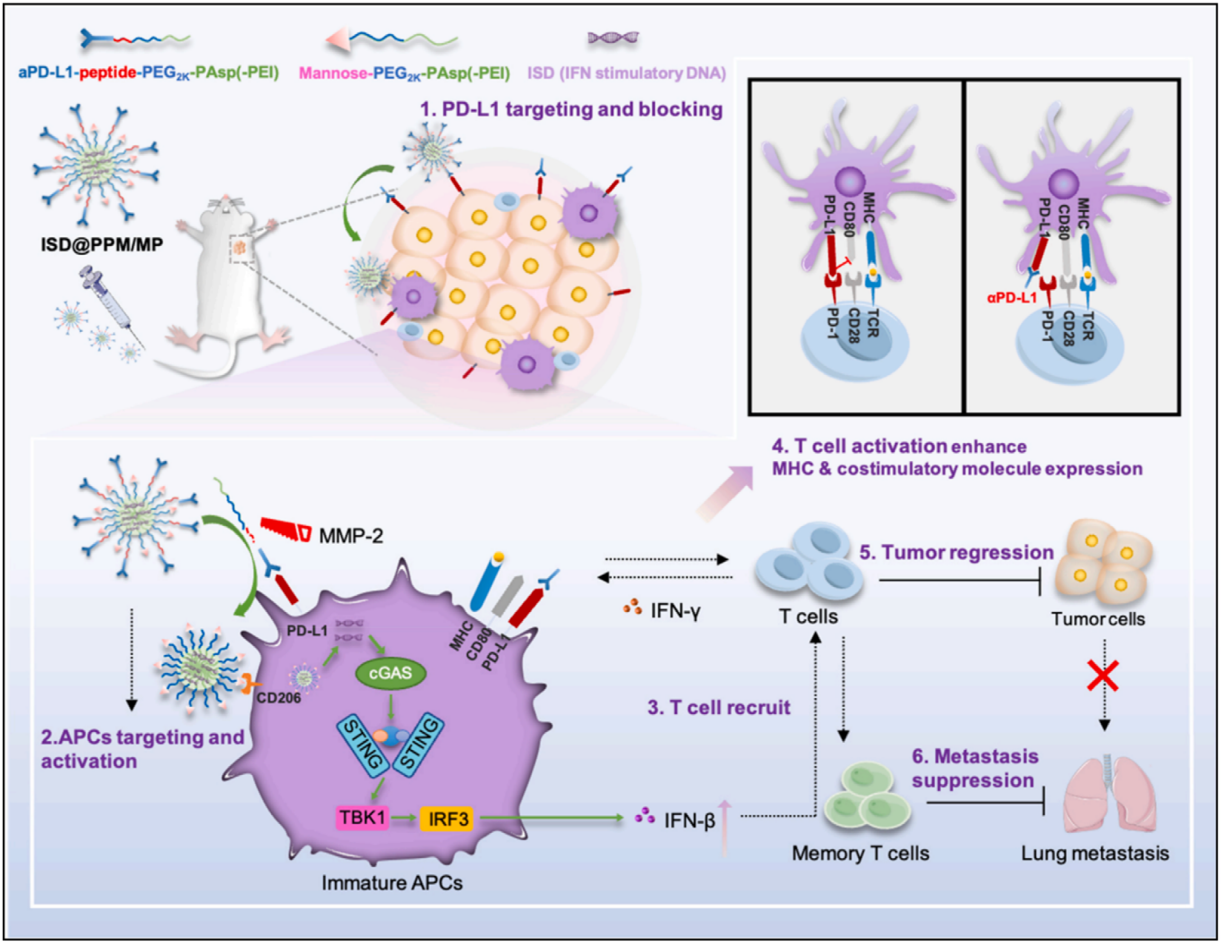
Schematic illustration of aPD‐L1‐/mannose‐targeting and MMP‐2‐sensitive nanodrug (ISD@PPM/MP) carrying ISD for immunotherapy. Reproduced with permission.^[^ ^73b^ ^]^ Copyright 2023, Elsevier.

Although considerable effort is still required to fully comprehend the optimal combination of STING agonists with ICBs, recent advancements in nanoparticle design are paving the way for more robust and targeted systemic immunotherapies. These innovations enable precise regulation over the release and activation of multiple therapeutic agents in a spatiotemporally controlled manner, as demonstrated by recent work reported by Shuai et al.^[^ ^73b^ ^]^ The authors developed a core‐shell nanoparticle with hierarchical tumor‐targeting capabilities to co‐deliver PD‐L1 antibodies (aPD‐L1) and the STING agonist interferon stimulatory DNA (ISD) (**Figure** **7**). The system was constructed using two triblock copolymers: Mal‐GPLGVRG‐PEG2k‐PAsp (PEI), featuring Mannose‐PEG2k‐PAsp (PEI) and an MMP‐2‐sensitive peptide linker. These copolymers co‐assembled into a mannose‐targeted nanocarrier, effectively encapsulating ISD in the core while conjugating aPD‐L1 to the surface through the MMP‐2 sensitive peptide.^[^ ^73b^ ^]^ This sophisticated design facilitated the sequential release of aPD‐L1 and the STING agonist, enabling a dual‐phase immune activation. ISD was first released to activate the STING signaling pathway in APCs, enhancing the recruitment and priming of T cells. The subsequent MMP‐2‐triggered release of aPD‐L1 reversed tumor‐induced immunosuppression by blocking the PD‐L1/PD‐1 axis, further amplifying T cell‐mediated antitumor responses. This programmed delivery strategy significantly inhibited tumor growth and metastasis in a murine orthotopic 4T1 breast cancer model, demonstrating the synergistic efficacy of combining STING agonists with ICB.^[^ ^73b^ ^]^

Besides the combination therapy of STING agonist‐based nanotherapeutics with ICBs regulating adaptive immune cells (particularly T cells) mentioned above, integrating STING agonists with immune checkpoint inhibitors targeting innate immune cells is another powerful approach to promote immunotherapy.^[^ ^74^ ^]^ A primary innate immune checkpoint of focus for combination regimens with STING agonists is signal regulatory protein α (SIRPα), an inhibitory immune checkpoint molecule expressed on myeloid cells, including neutrophils, macrophages, and DCs.^[^ ^75^ ^]^ SIRPα recognizes CD47 as its ligand, an antiphagocytic factor expressed on the surface of nearly all cells and often overexpressed on tumor cells, serving as a “don't eat me” signal that helps cancer cells escape phagocytosis by myeloid cells.^[^ ^74^, ^76^ ^]^ Moreover, elevated CD47 expression has been correlated with poor prognosis in various cancers.^[^ ^77^ ^]^ These findings provide a strong rationale that CD47 is a promising therapeutic target for antitumor immunotherapy. Indeed, several preclinical and clinical studies targeting the CD47‐SIRPα pathway in both hematologic and solid tumors are currently undergoing.^[^ ^78^ ^]^ However, in the majority of situations, blockade of the SIRPα‐CD47 interaction using anti‐CD47 antibodies alone may be inadequate to trigger significant phagocytosis and antitumor immunity. Therefore, combining immune activators, such as STING agonists, is crucial to enhance the therapeutic efficacy of CD47‐SIRPα blockade.

Notably, recent findings revealed that STING signaling is essential for the antitumor effects of CD47 blockade therapy, as both anti‐CD47‐triggered DCs cross‐priming of CD8^+^ T cells and the induction of type I interferons after anti‐CD47 treatment depended on STING activation.^[^ ^79^ ^]^ Furthermore, studies led by Ohkuri and co‐workers provided compelling evidence that intratumoral injection of cGAMP could overcome the limitations of anti‐CD47 monotherapy via promoting inflammatory cytokines production and recruiting M1‐like macrophages/monocytes to the TME.^[^ ^80^ ^]^ They demonstrated that the combination of cGAMP with antagonistic anti‐CD47 mAb significantly enhanced the phagocytic ability of macrophages/monocytes to engulf tumor cells and triggered systemic antitumor immune responses.^[^ ^80^ ^]^ Taken together, these findings have clarified the underlying mechanisms of combination therapy with STING agonists and anti‐CD47 antibodies, which can be summarized as follows: i) The antitumor response and therapeutic efficacy of CD47 blockade depend on the tumor infiltration of macrophages/monocytes, which is facilitated by STING signaling activation.^[^ ^80a^ ^]^ ii) In parallel, the blockade of the SIRPα‐CD47 interaction within the TME enhances the STING agonist‐mediated antitumor immunity by facilitating tumor cell phagocytosis and activation of tumor‐reactive T cells.^[^ ^80a^ ^]^

**Figure 8:**
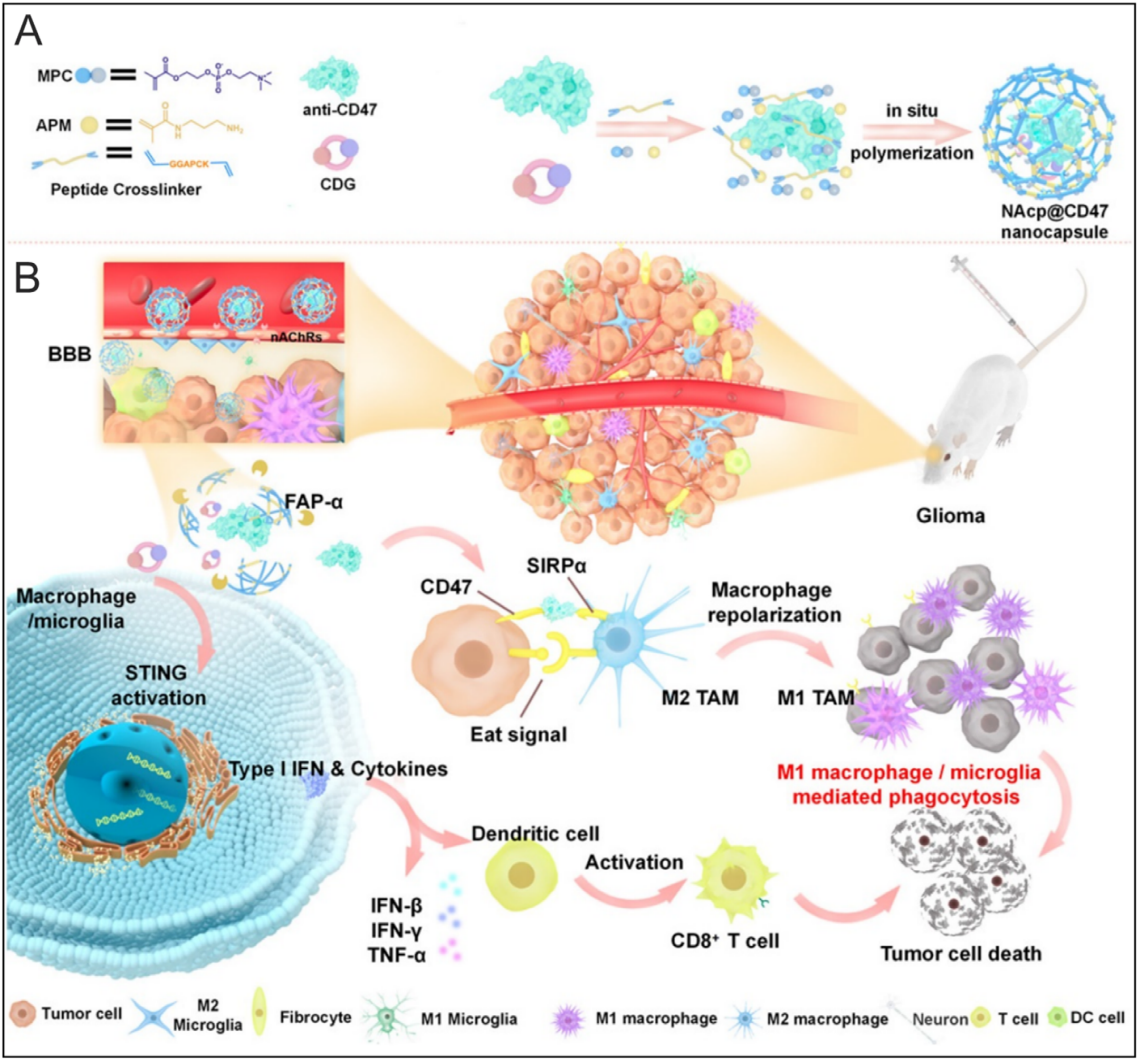
Schematic illustration of the NAcp@CD47 nanocapsule for dual delivery of anti‐CD47 antibodies and STING agonists for GBM immunotherapy by repolarization of macrophages and promotion of phagocytosis. (A) The process of constructing the FAP‐α responsive prodrug nanocapsule: anti‐CD47 antibodies and CDG are co‐encapsulated in NAcp@CD47 using MPC, APM, and FAP‐α‐responsive peptide crosslinker in situ polymerization nanocapsule. (B) The mechanism of NAcp@CD47 polarization of microglia and macrophages against GBM. After intravenous injection, NAcp@CD47 can be delivered to the CNS, crossing the BBB mediated by MPC, releasing anti‐CD47 antibodies and CDG after FAP‐α enzymatic degradation in the GBM microenvironment to block the phagocytosis checkpoint CD47‐SIRPα and promote the production of IFNs via the STING signaling pathway. Consequently, the reprogrammed microglia (or macrophages) enhance the phagocytosis of cancer cells. Similarly, IFNs facilitate increased infiltration of immune cells and increase immunogenicity to convert “cold” tumors into “hot” tumors. Reproduced with permission.^[^ ^81^ ^]^ Copyright 2022, Ivyspring International Publisher.

The above facts provide theoretical support for researchers to design a combination regimen of STING agonists with CD47 blockade to promote potent antitumor immunity. For instance, Wang et al. developed a dual‐loaded BBB‐permeable nanocapsule called NAcp@CD47, in which the STING agonist CDG (cyclic‐di‐GMP) and anti‐CD47 antibodies were co‐encapsulated by a thin polymer layer formed via in situ free‐radical polymerization of N‐(3‐aminopropyl) methacrylamide, 2‐methacryloyloxyethyl phosphorylcholine, and FAP‐α‐responsive protease‐degradable crosslinkers (**Figure** **8**).^[^ ^81^ ^]^ After intravenous injection, NAcp@CD47 displayed remarkable tumor inhibition, increased polarization of M1‐like macrophages, enhanced phagocytosis, and improved T cell infiltration in the TME in both subcutaneously implanted and orthotopic glioblastoma mouse models. In contrast, anti‐CD47 antibody treatment alone could not achieve the same therapeutic potency, highlighting the importance of the synergistic modulation of STING agonists and innate immune checkpoint inhibitors through NAcp@CD47.^[^ ^81^ ^]^

Overall, these findings underscore the synergistic potential of rationally designed combination regimens involving STING agonists and ICBs to achieve durable and robust antitumor responses, particularly in tumors resistant to monotherapy.

#### 3.3.2 STING Agonists‐Based Nanotherapeutics Combined with IDO Inhibitors

In recent years, the indoleamine‐2,3 dioxygenase‐1 (IDO1) enzyme, responsible for the degradation of the essential amino acid tryptophan (Trp),^[^ ^82^ ^]^ has emerged as a promising target in cancer immunotherapy due to its pivotal role in tumor immune evasion.^[^ ^83^ ^]^ Comprehensive reviews on the intricate relationship between Trp catabolism and cancer immunity have been previously provided by Platten^[^ ^84^ ^]^ and Lemos.^[^ ^85^ ^]^ Biochemically, IDO1 is a key metabolic enzyme that catalyzes the rate‐limiting step of Trp catabolism, resulting in the production of kynurenine (Kyn) and downstream metabolites, collectively termed the Kyn pathway.^[^ ^84^ ^]^ IDO1 activation drives extensive Trp catabolism, depriving effector T cells of Trp and resulting in their starvation within the TME.^[^ ^84^, ^86^ ^]^ This metabolic disruption impairs T cell function and compromises the antitumor immune responses. Additionally, the tryptophan metabolites, predominantly Kyn, promote the activation and immunosuppressive function of Tregs, further dampening antitumor immune responses.^[^ ^86a^ ^]^

Beyond its role in T cells, IDO1 is often overexpressed by APCs in various cancers, such as DCs and macrophages, where its activation drives these cells towards an immunosuppressive phenotype that fosters immune tolerance.^[^ ^86a^ ^]^ Clinically, increased IDO expression has been correlated with poor prognosis in cancers such as lung cancer, colorectal cancer, and melanoma.^[^ ^87^ ^]^ However, in certain cancers, IDO expression can be triggered by inflammation and increased T‐cell infiltration,^[^ ^86^, ^88^ ^]^ where its upregulation may signify a strong spontaneous antitumor immunity and correlate with a more favorable prognosis.^[^ ^89^ ^]^

The broad expression and interferon‐inducible nature of IDO1 are critical to understanding how inflammation and T cell infiltration regulate its expression within the TME. As a counter‐regulatory mechanism, IDO1 is crucial for maintaining immune homeostasis and tolerance. Its expression is highly inducible and is typically absent unless triggered by inflammatory signals or T cell activation within the TME. Recent studies reveal that innate immune responses during tumorigenesis or T cell‐mediated activation, including those triggered by immunotherapy, can upregulate IDO1.^[^ ^86a^ ^]^

Consistent with the concept that STING/IFN‐I signaling plays a critical role in immune regulation, STING agonists are also strong inducers of IDO, which may contribute to immune tolerance in certain tumor settings. This paradoxical effect is particularly evident in tumors with low antigenicity, where STING agonist treatment promotes IDO‐mediated immunosuppressive pathways, ultimately dampening antitumor immunity.^[^ ^90^ ^]^ For instance, studies in a Lewis lung carcinoma (LLC) mouse model demonstrated that STING‐deficient mice exhibited slower tumor growth when challenged with native LLC tumors, compared to tumors expressing neoantigens.^[^ ^90^ ^]^ These findings may help elucidate why STING agonists demonstrate variable therapeutic efficacy across different cancer types.

Given this evidence, the potential upregulation of IDO by STING activation holds critical implications for cancer immunotherapy. Poor therapeutic responses to STING agonist treatments in some cases may arise in part from the elevated IDO activity induced by STING/IFN‐I signaling or activated T cells, which suppress the desired antitumor immune effects.^[^ ^86^, ^88^ ^]^ This suggests that combining STING agonists with IDO inhibitors may offer a more effective therapeutic approach. Additionally, in tumors with low immunogenicity, IDO‐driven immune tolerance may limit the efficacy of STING‐based monotherapy.^[^ ^86^, ^90^ ^]^ Collectively, these considerations suggest that combining STING agonists with IDO inhibitors could help overcome these limitations. The strong correlation between STING/IFN‐I signaling and IDO expression further strengthens the rationale for testing this combination in clinical settings.

Based on these findings, efforts have been made to explore the synergistic potential of combining STING agonists with IDO inhibitors in cancer immunotherapy.^[^ ^91^ ^]^ For instance, Syeda and colleagues developed a prodrug nanoplatform to enhance these combinatorial effects.^[^ ^91b^ ^]^ They synthesized an esterase‐responsive prodrug by conjugating the STING agonist MSA‐2 to the IDO inhibitor NLG‐919 through an ester bond. The yielded prodrug, referred to as MSA‐NLG, self‐assembled into nanoparticles with the surface modification of DSPE‐mPEG2K.^[^ ^91b^ ^]^ After intravenous administration, these nanoparticles dissociated within the TME through esterase hydrolysis, releasing MSA‐2 and NLG‐919. The prodrug nanoparticles facilitated the recruitment and activation of DCs and CD8+ T cells, decreased myeloid‐derived suppressor cells (MDSCs), and achieved more potent antitumor immunity compared to either treatment alone in xenograft tumor models of C57BL/6 mice.^[^ ^91b^ ^]^ These results highlight the synergistic potential of co‐delivering STING agonists and IDO inhibitors, providing a rational basis for designing combination regimens that coordinate STING activation with IDO blockade to maximize therapeutic efficacy and safety.

#### 3.3.3 STING Agonists‐Based Nanotherapeutics Combined with Cancer Vaccine

Cancer therapeutic vaccines hold significant potential in cancer immunotherapy via stimulating innate and/or adaptive antitumor immune responses while alleviating immunosuppression within the TME. However, the clinical efficacy of conventional peptide‐based subunit vaccines has been limited, often showing only marginal benefits in patients.^[^ ^92^ ^]^ This limitation is largely due to the poor immunogenicity of peptide antigens, which fail to trigger robust antigen‐specific effector T‐cell responses.^[^ ^93^ ^]^ The weak immunogenicity of peptide antigens arises from several intertwined factors, including their rapid clearance, insufficient uptake by DCs, inadequate accumulation in lymph nodes, and inefficient cross‐presentation on MHC‐I molecules.^[^ ^94^ ^]^ Additionally, the inappropriate selection of immunostimulatory adjuvants exacerbates these limitations, further hindering the activation of robust antigen‐specific immune responses.^[^ ^92^, ^93^, ^95^ ^]^ Although several clinical trials of neoantigen‐targeted cancer vaccines have demonstrated their ability to induce detectable antigen‐specific T cell responses, these responses are often suboptimal, with insufficient CD8+ T cell activation and limited therapeutic efficacy.^[^ ^96^ ^]^ Even when sufficient antitumor CD8+ T cells are generated in the systemic circulation, their intratumoral trafficking remains inefficient,^[^ ^97^ ^]^ thereby limiting therapeutic outcomes. A major contributing factor to these shortcomings is the inefficient co‐delivery of antigenic peptides and immunostimulatory adjuvants to draining lymph nodes, resulting in immunological tolerance and weakened CD8+ T cell responses.^[^ ^98^ ^]^ To overcome these challenges, integrating potent immunostimulants such as STING agonists as adjuvants for peptide vaccines, combined with the co‐delivery of antigens and adjuvants in multimeric formats (e.g., nanoparticles or liposomes),^[^ ^94^, ^99^ ^]^ represents a promising strategy to enhance innate immunity, improve antigen immunogenicity, and boost vaccine efficacy.

Recent studies have increasingly focused on the co‐delivery of STING agonists and peptide antigens using nanotechnology, exploring the synergistic potential of nanoadjuvants to enhance STING pathway activation and tumor vaccine efficacy.^[^ ^93^, ^100^ ^]^ These approaches leverage advanced nanoplatforms to address immunogenicity and delivery efficiency challenges, offering new avenues for cancer immunotherapy. One representative example is the work by Wilson and colleagues, who developed pH‐responsive, endosomal membrane‐destabilizing polymersomes leveraging stimuli‐responsive poly(methacrylates) to facilitate efficient cellular internalization and cytosolic release of CDN for STING pathway activation.^[^ ^40^ ^]^ These endosomolytic polymersomes were employed to co‐deliver the STING agonist cGAMP alongside peptide antigens, including tumor neoantigens, to construct a cancer therapeutic vaccine termed nanoSTING‐vax.^[^ ^100a^ ^]^ This system enabled simultaneous uptake of peptide antigens and cGAMP by APCs, promoting their release into the cytosol via endosomal escape. Consequently, nanoSTING‐vax significantly enhanced MHC‐I antigen presentation, boosted DC maturation, and triggered robust antigen‐specific CD8+ T‐cell priming, leading to effective tumor suppression in multiple murine models.^[^ ^100a^ ^]^

In a follow‐up study, the authors further optimized this nanoparticle‐based vaccine platform by incorporating two synergistic adjuvants (i.e., STING agonist cGAMP and TLR4 agonist MPLA) at defined ratios using impingement jet mixing technology.^[^ ^93^ ^]^ The resulting uniform pH‐responsive polymersomes induced potent IFN‐I responses, promoted DCs activation, and strengthened antigen‐specific CD8+ T cell responses. These immunological enhancements translated into notable tumor growth inhibition and prolonged survival across multiple preclinical models.^[^ ^93^ ^]^

**Figure 9:**
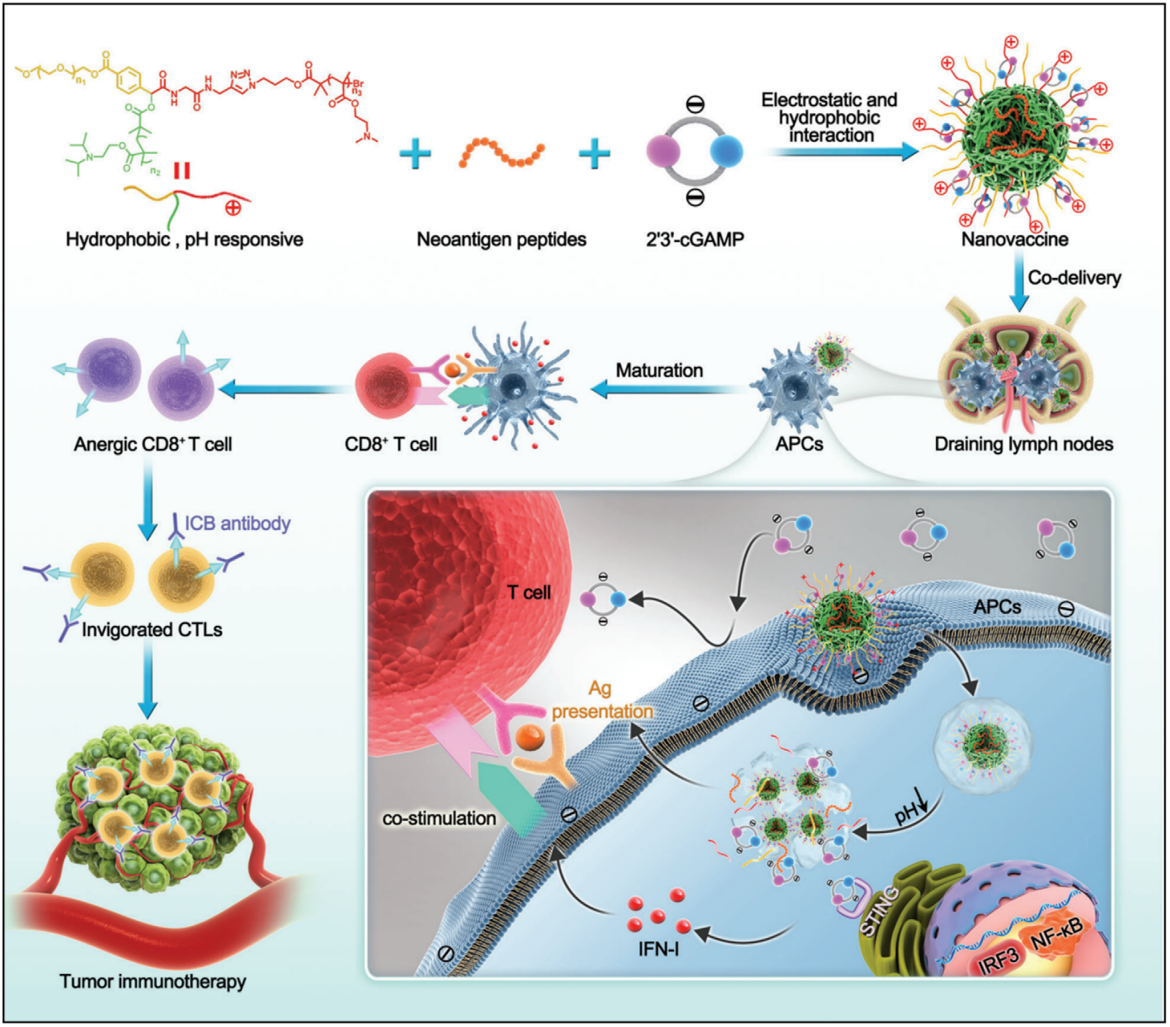
Schematic illustration of pH‐responsive multivesicular polymeric nanovaccines (NVs) for the codelivery of STING agonists and neoantigens in combination with tumor immunotherapy. The star‐shaped polymers were self‐assembled into pH‐responsive nanoparticles (NPs) that coloaded cGAMP and neoantigen peptides through electrostatic and hydrophobic interactions, respectively. The NVs efficiently codelivered cGAMP and neoantigens to the draining lymph nodesand intranodal APCs. Upon cell internalization by endocytosis, NVs were disassembled in response to the acidity in the endosome and escaped to the cytosol for STING activation by cGAMP and neoantigen presentation by MHC. NVs elicited sustained antigen presentation, potentiated and prolonged the neoantigen‐specific T‐cell responses, and ameliorated the tumor immuno‐suppression in the TME. As a result, the combination of these NVs withICB showed robust tumor therapeutic efficacy in multiple tumor models. Reproduced with permission.^[^ ^100b^ ^]^ Copyright 2022, John Wiley & Sons.

Complementing these efforts, Zhu et al. developed a pH‐responsive polymeric nanovaccine platform for the co‐delivery of cGAMP and peptide neoantigens (**Figure** **9**).^[^ ^100b^ ^]^ This system was tested with model antigens, including ovalbumin (OVA), Adpgk (ASMTNMELM neoantigen), and the E7 antigen (derived from TC‐1 carcinoma expressing human papillomavirus oncoviral). Subcutaneous administration of these nanovaccines in syngeneic mouse models of EG7.OVA, TC‐1, and MC38 tumors resulted in significantly improved tumor growth control.^[^ ^100b^ ^]^ Mechanistically, the combinatorial efficacy of STING agonist and peptide antigens was associated with enhanced antigen‐specific CD8+ T cells expansion and elevated intratumoral IFN‐γ levels in peripheral blood of mice treated with cGAMP/antigen‐loaded nanovaccines. Notably, mice with long‐term survival rejected a flank tumor cell rechallenge, indicating durable immune memory.^[^ ^100b^ ^]^

To explore additional delivery strategies, Yu et al., developed an acid‐sensitive nanoplatform to enable the intracellular co‐delivery of the STING agonist MSA‐2 and antigen peptides.^[^ ^100d^ ^]^ This approach leveraged iron oxide nanoparticles (IONPs), which enhanced STING‐mediated IFN‐I responses by promoting intracellular ROS generation and activating the NF‐κB pathway.^[^ ^100d^ ^]^ Utilizing reversible addition‐fragmentation chain‐transfer polymerization, the researchers synthesized acid‐ionizable copolymers with exceptional pH sensitivity and endosomolytic properties. These copolymers were co‐assembled with IONPs and MSA‐2 via electrostatic interactions to form a nanoadjuvant system, which was subsequently mixed with OVA antigen or autologous tumor antigen to create a nanovaccine (PEIM@antigen).^[^ ^100d^ ^]^ The resulting iron nanoadjuvant (PEIM) effectively delivered model antigen OVA to CD169+ APCs and significantly promoted antigen cross‐presentation, leading to a 55‐fold increase in antigen‐specific CD8+ T cells compared to the soluble antigen administration. Immunization with the PEIM@OVA nanovaccine effectively prevented lung metastasis and eradicated tumors in MC38 colorectal and B16‐OVA melanoma cancer models.^[^ ^100d^ ^]^

Expanding the application of polymeric systems, Li et al. designed and synthesized a polymeric prodrug nanoplatform integrating a diblock copolymer with pH‐responsive properties and the STING agonist DMXAA.^[^ ^100e^ ^]^ The amphiphilic diblock copolymer, poly(ethylene glycol)‐block‐poly(2‐(diisopropanol amino) ethyl methacrylate) (PEG‐b‐PDPA), was synthesized via reversible addition‐fragmentation chain transfer polymerization. To enable STING activation, DMXAA was covalently conjugated to the PEG‐b‐PDPA backbone through an ester bond, forming the DMXAA‐grafted diblock copolymer (PEG‐b‐PDPA‐DMXAA).^[^ ^100e^ ^]^ To enhance endosome escape, the cationic polymer 1,2‐epoxytetradecane alkylated oligoethylenemine 800 (OEI‐C14) was selected to co‐assemble with PEG‐b‐PDPA‐DMXAA, leading to the formation of micellar nanoparticles. Subsequently, the antigen OVA was encapsulated using a nanoprecipitation approach, achieving dual‐delivery of STING agonist and peptide antigens within the same nanocarrier.^[^ ^100e^ ^]^ This polymeric prodrug vaccine platform facilitated synchronous delivery of neoantigens and DMXAA to DCs, effectively augmenting antigen presentation via STING activation. The system potentiated type I IFN secretion, enhanced antigen‐specific CD8+ T cell responses, and significantly suppressed tumor growth in murine models of 4T1 breast cancer and B16‐OVA melanoma.^[^ ^100e^ ^]^

In a complementary study, Liu et al. developed self‐degradable nanoparticles composed of poly(β‐amino ester)s (PBAEs) to co‐deliver the STING agonist 2′3’‐cGAMP and the OVA antigen for B16F10‐OVA melanoma immunotherapy.^[^ ^100c^ ^]^ PBAEs were selected due to their biocompatibility, structural versatility, ability to bind nucleic acids electrostatically, and intrinsic endosomolytic properties, making them highly suitable for gene and drug delivery applications. In this study, mPEG_44_‐b‐P(O_x_PH)_126_ was synthesized through ring‐opening polymerization of N‐Boc‐1,4‐oxazepan‐7‐one (O_x_P_Boc_). The OVA and cGAMP were encapsulated into the polymer matrix via electrostatic interactions, yielding nanoparticles with uniform size and high drug‐loading capacity.^[^ ^100c^ ^]^ Following intratumoral administration, the nanovaccine rapidly drained into sentinel lymph nodes (LNs) and was efficiently internalized by APCs at both LNs and tumor sites. The combined activity of STING agonist‐mediated immune stimulation and OVA antigen release promoted the expression of IFN‐β, TNF‐α, and chemokines CXCL9 and CXCL10.^[^ ^100c^ ^]^ This resulted in the recruitment and activation of CD8+ T lymphocytes, effectively inhibiting tumor progression and prolonging survival in murine B16 F10‐OVA subcutaneousmelanoma models. These results further underscore the potential of self‐degradable polymeric nanocarriers for co‐delivery strategies in cancer immunotherapy.

Additionally, Xu et al. developed a manganese‐silica nanoplatform for the co‐delivery of tumor neoantigen peptides, STING agonist CDN, and Mn^2+^ adjuvants, aiming to enhance synergetic cancer immunotherapy.^[^ ^100f^ ^]^ The nanoplatform was engineered by synthesizing hollow mesoporous silica nanoparticles (HMSNs) with large pore sizes, which were surface‐coated with manganese oxide (MnO_x_) nanoparticles. To further optimize delivery, MnO_x_@HMSN nanoparticles were surface‐functionalized with amine groups and co‐loaded with CDN and antigen peptides.^[^ ^100f^ ^]^ The synergy between the co‐delivered neoantigen peptides, CDNs, and Mn^2+^ adjuvants was demonstrated in murine models of CT26 and MC38 colon carcinoma. Intratumoral injection of MnO_x_@HMSN(CDA+AH1) significantly increased the proportion of AH1‐specific CD8α+ T‐cells in mice with bilateral CT26 tumors, compared to those treated with a soluble vaccine formulation. Furthermore, MnO_x_@HMSN(CDA+AH1) treatment substantially suppressed the growth of both primary and distant tumors, whereas soluble formulations showed no antitumor activity.^[^ ^100f^ ^]^ The platform was also applied to another tumor‐specific neoantigen Adpgk, derived from MC38 colorectal carcinoma. In a bilateral MC38 tumor model, treatment with MnO_x_@HMSN (CDA+Adpgk) outperformed a soluble mixture of CDA, Adpgk, and Mn^2+^, demonstrating potent local and abscopal antitumor effects.^[^ ^100f^ ^]^ These findings highlight MnO_x_@HMSN as a robust nanoplatform for enhancing the efficacy of CDN‐based cancer vaccines, offering a robust strategy to amplify immune responses and improve therapeutic outcomes.

In summary, recent advancements in nanotechnology‐enabled vaccine platforms have demonstrated remarkable potential in overcoming critical barriers in cancer immunotherapy by enabling the precise co‐delivery of peptide antigens and STING agonists. These platforms, including pH‐responsive polymersomes, acid‐sensitive nanoparticles, polymeric prodrug systems, self‐degradable nanoparticles, and manganese‐silica nanoplatforms, have shown promising results in enhancing STING pathway activation, boosting antigen‐specific CD8+ T cell responses, and achieving robust tumor control across multiple preclinical models. Together, these studies highlight the transformative potential of nanotechnology in developing next‐generation cancer vaccines, paving the way for future clinical translation.

#### 3.3.4 STING Agonists‐Based Nanotherapeutics Combined with ACT

ACT is an emerging immunotherapeutic strategy that involves the in vitro expansion and genetic engineering of autologous or allogeneic immune cells, which are then reinfused into the patient to mediate antitumor, anti‐inflammatory, or antiviral effects.^[^ ^101^ ^]^ ACT utilizes various immune cell types, such as T cells, NK cells, and tumor‐infiltrating lymphocytes. It also employs techniques such as CAR modification or gene‐modified T cells expressing novel T‐cell receptors (TCR) to enhance the specificity and efficacy of the immune response.^[^ ^102^ ^]^

ACT has shown remarkable efficacy in treating advanced hematological cancers. Among these, CAR‐T cell therapy stands out as the most well‐established approach, achieving high rates of complete remission in patients with relapsed, refractory, or treatment‐resistant B‐cell malignancies, and more recently, in multiple myeloma. These groundbreaking clinical successes have led to the FDA approval of several CAR‐T cell cancer therapies, with many promising clinical trials of similar approaches currently underway.^[^ ^103^ ^]^

However, the translation of ACT to solid tumors remains a significant challenge.^[^ ^104^ ^]^ The main obstacles posed by solid tumors are target antigen selection, genetic characteristics, and immunosuppressive TME. On the one hand, the antigenic heterogeneity of solid tumors complicates the design of CARs capable of effectively targeting diverse tumor cells within a specific tumor type. This challenge undermines the immunological targeting of solid tumors and contributes to the limited clinical efficacy of ACT. On the other hand, insufficient lymphocyte trafficking to solid tumor sites further hampers the efficacy of ACT, as the current cell delivery method depends on systemic infusion of the transferred cells. More critically, solid tumors create an immunosuppressive TME characterized by the expression of inhibitory receptors (e.g., PD‐L1) and the secretion of immunosuppressive cytokines (e.g., TGF β and IL‐10). These factors inhibit T cell expansion and function, thereby suppressing T cell responses.^[^ ^104b^ ^]^ Furthermore, CAR T cell infiltration into the tumor can lead to the upregulation of inhibitory molecules on the surface of CAR T cells and other immune cells within the TME,^[^ ^104^, ^105^ ^]^ while also promoting Treg cells accumulation, which exacerbates immunosuppression. To address these challenges, combination therapies involving STING agonists offer a promising strategy to address the limitations of ACT in solid tumors and improve clinical outcomes.

In line with this, preclinical studies combining STING agonists with CAR T cell therapies were recently reported.^[^ ^106^ ^]^ Serody and colleagues demonstrated that STING agonists, such as DMXAA and cGAMP, promoted the trafficking and persistence of T helper 17 (Th17) and CD8+ T 17 (Tc17) CAR T cells in the TME, enhancing the antitumor effects of CAR T cell therapy in an orthotopic advanced murine breast cancer model.^[^ ^106a^ ^]^ In another study, June and colleagues found that the cGAMP analogue IMSA101 modulated the TME by promoting a pro‐inflammatory cytokine environment, improving the infiltration of CAR T cells and other immune cells into the tumor. They also demonstrated that IMSA101 treatment facilitated CAR T cell function through STING agonist‐induced IL‐18 secretion in mouse models of pancreatic ductal adenocarcinoma and melanoma.^[^ ^106b^ ^]^ This study offers a mechanistic understanding of how STING agonists enhance CAR T cell activity and reinforces the potential for advancing combination therapies involving STING agonists and CAR T cells in clinical settings.

Apart from these mechanistic interpretations of the joint action of STING agonist and CAR T cell therapy, the preclinical effects of this combined use, integrating nanotechnology for solid tumor control, were reported by Stephan and colleagues.^[^ ^107^ ^]^ The authors designed and constructed a microporous scaffold to enable efficient localized delivery of tumor‐targeting T cells and the STING agonist c‐di‐GMP. This porous scaffold matrix was composed of polymerized alginate conjugated with collagen‐mimetic GFOGER peptides via carbodiimide chemistry, naturally facilitating lymphocyte binding and migration. Mesoporous silica microparticles were embedded within this matrix, loaded with the STING agonist c‐di‐GMP, and functionalized to display the immunostimulatory signals, including anti‐CD3, anti‐CD28, and anti‐CD137 antibodies for T cells.^[^ ^107^ ^]^ When implanted into a surgical cavity, the scaffold enabled substantial T cell expansion and migration to the tumor resection site and tumor‐draining lymph nodes, effectively eliminating residual tumor cells and reducing the risk of relapse. Moreover, STING activation by c‐di‐GMP generated a localized immunostimulatory environment that promoted T cell priming and activation, including the de novo activation of endogenous T cells able to attack tumor cells beyond CAR T cell recognition. Consequently, this scaffold‐mediated co‐delivery of tumor‐specific CAR T cells along with c‐di‐GMP triggered robust antitumor immune responses in both pancreatic and melanoma models and led to sustained immunity against tumor rechallenge.^[^ ^107^ ^]^ These studies established a foundation for combining STING agonists with CAR T cell therapy, providing preclinical evidence to support the exploration of this synergistic approach in solid tumor treatment for clinical settings.

### 3.4 STING Agonists‐Based Nanotherapeutics Combined with Phototherapy

Phototherapy, which includes photothermal therapy (PTT) and photodynamic therapy (PDT), has gained attention as a promising cancer treatment modality. A growing body of evidence demonstrates that phototherapy not only induces direct cytotoxicity by ablating irradiated cancer cells but also triggers an antitumor immune response. However, single‐modal therapies based on PTT or PDT often fail to elicit a strong and sustained immune response, limiting their therapeutic efficacy. To address these limitations, a variety of combination therapeutic strategies have been explored, such as combined phototherapy/vaccine, phototherapy/immunoadjuvant, and phototherapy/ICBs, aiming to compensate for the deficiencies of a single photo‐immunotherapy in cancer treatment. This section focuses on the recent advances in cancer photo‐immunotherapy, specifically combining STING agonists‐based nanotherapeutics with PTT or PDT. Furthermore, the underlying mechanisms behind these combination regimens will also be discussed, laying the groundwork for their clinical translation.

#### 3.4.1 STING Agonists‐Based Nanotherapeutics Combined with PTT

PTT, which employs photothermal conversion agents to induce localized hyperthermia under near‐infrared (NIR) light irradiation, has emerged as a promising approach for cancer treatment.^[^ ^108^ ^]^ PTT offers advantages such as non‐invasiveness, low systemic toxicity, and high selectivity. It exerts direct tumoricidal effects by causing DNA damage, protein denaturation, and cellular membrane disruption.^[^ ^109^ ^]^ Furthermore, PTT promotes antitumor immune activation via several mechanisms, including the release of TAAs, DAMPs, and immune‐stimulatory cytokines from thermally ablated tumor cells. It also enhances the expression of heat shock proteins and facilitates lymphocyte migration towards the tissues experiencing mild hyperthermia.^[^ ^108^, ^110^ ^]^

Despite its potential, the therapeutic effectiveness of PTT in treating solid tumors, particularly larger or deeply situated ones, is hampered by the restricted tissue penetration depth of NIR light and the inadequate intratumoral accumulation of photothermal agents,^[^ ^111^ ^]^ often resulting in incomplete tumor eradication. Additionally, PTT's localized effects are insufficient for controlling the growth of distant tumors or metastases.^[^ ^112^ ^]^ This limitation is further compounded by the immunosuppressive TME, which is exacerbated by PTT‐induced hyperthermia, leading to the expansion and accumulation of immunosuppressive MDSCs.^[^ ^113^ ^]^ Consequently, immune responses elicited by PTT alone are generally weak and predominantly immunosuppressive. These challenges underscore the need to combine PTT with immune‐stimulating agents to synergistically enhance antitumor immunity for treating established solid tumors and distant metastases.

The rationale for combining PTT with STING agonists lies in their complementary mechanisms. PTT‐induced hyperthermia facilitates the release of TAAs, DAMPs, and pro‐inflammatory signals, which are pivotal in bolstering the immune‐stimulating efficacy of STING agonists. PTT also induces ICD, activating and amplifying the STING pathway. Conversely, STING agonists can overcome the localized nature of PTT by inducing robust systemic immune responses, thus improving PTT's efficacy against both primary and metastatic tumors. Together, these mechanisms form a strong foundation for the development of combinational therapeutic strategies of STING agonists with PTT.

Recent studies have demonstrated promising progress in combining PTT with STING agonists to achieve synergistic therapeutic effects. This combination offers a practical approach to augment antitumor immune responses, and its efficacy has been validated in several preclinical studies.^[^ ^114^ ^]^ For instance, Ma et al. engineered a multifunctional photoimmunothernostic nanomedicine for the combination of mild PTT and STING agonist‐based immunotherapy.^[^ ^114a^ ^]^ The nanoparticle was prepared by co‐assembling the synthetic phototheranostic agent croconaine dye IR1024, the STING agonist diABZI, and DSPE‐PEG‐bis‐5HT. The resulting nanoparticles, denoted SAPTNs, had a diameter of about 121.4 nm and a surface charge of ‐1.32 mV. IR1024 enabled NIR fluorescence and photoacoustic imaging of tumor‐bearing mice and could be activated by NIR‐II laser to recruit neutrophils into the TME. These neutrophils secreted myeloperoxidase, which facilitated the production of radical bis‐5‐hydroxytryptamine on the surface of SAPTNs.^[^ ^114a^ ^]^ The radicals bound to laminin in the extracellular matrix and gradually quenched, promoting SAPTNs’ anchoring to Tyr‐based proteins in the TME, thus enabling the sustained release of diABZI and activation of STING signaling. Intravenous injection of SAPTNs led to 100% tumor eradication and significantly extended survival with no detectable tumors for at least 2 months in an orthotopic breast cancer model, compared to the individual agents. Furthermore, intratumoral injection of SAPTNs resulted in an abscopal effect, suppressing the growth of distant solid tumors.^[^ ^114a^ ^]^ This innovative nanoplatform highlights the powerful synergy between PTT and STING agonists, offering a promising strategy to enhance antitumor immunity and therapeutic efficacy.

Similarly, Sun and colleagues developed a biomimetic nanoplatform, termed CMM‐DiR, using a cancer cell membrane to co‐encapsulate the photothermal agent (DiR) and manganese dioxide nanoparticles (MnO_2_ NPs) for in situ immunization.^[^ ^114b^ ^]^ In this system, the DiR‐mediated photothermal effect facilitated the release of TAAs, while manganese ions served as immune adjuvants to activate the STING pathway. The nanoplatform could be degraded quickly in the mildly acidic and high H_2_O_2_ conditions characteristic of the TME, resulting in a burst release of Mn^2+^ from CMM‐DiR.^[^ ^114b^ ^]^ After systemic administration, the Mn^2+^ coupled with targeted PTT elicited robust systemic antitumor immunity and enabled a durable tumor regression in multiple murine models, including primary, multinodular, recurrent, and metastatic B16‐F10 melanoma.^[^ ^114b^ ^]^ These findings highlight the significant therapeutic potential of combining PTT with STING agonists to combat advanced and metastatic tumors effectively.

In addition to the aforementioned croconaine and Dir, the combination of STING agonist with other photothermal conversion agents has also been investigated.^[^ ^114c–e^ ^]^ For instance, Zheng and colleagues designed a versatile nanoplatform co‐delivering photothermal sensitizer and manganese ions to synergistically enhance immune activation.^[^ ^114c^ ^]^ This nanoplatform was constructed by doping manganese ions (Mn^2+^) into Prussian blue (PB) to form Mn‐doped PB NPs (MnPB), followed by surface modification with MnO_x_ via a redox reaction between potassium permanganate and polyvinylpyrrolidone‐coated MnPB.^[^ ^114c^ ^]^ The final MnPB‐MnO_x_ nanoparticles had an average diameter of ≈168.5 ± 3.1 nm. Mechanistically, PB‐mediated PTT and MnO_x_‐catalyzed ROS generation collectively facilitated the release of TAAs. Simultaneously, the Mn^2+^ released from MnPB‐MnO_x_ degradation in the TME activated the STING pathway, further amplifying systemic immune responses.^[^ ^114c^ ^]^ The synergistic therapeutic efficacy of this nanoplatform was validated in both subcutaneous and bilateral orthotopic 4T1 tumor‐bearing mouse models, where increased tumor infiltration of M1 macrophages, CD8+ T cells, and NK cells was observed.^[^ ^114c^ ^]^ These results highlight the effective activation of both innate and adaptive immunity, underscoring the potential of combining STING agonists with Prussian blue‐mediated PTT for advanced cancer treatment.

**Figure 10:**
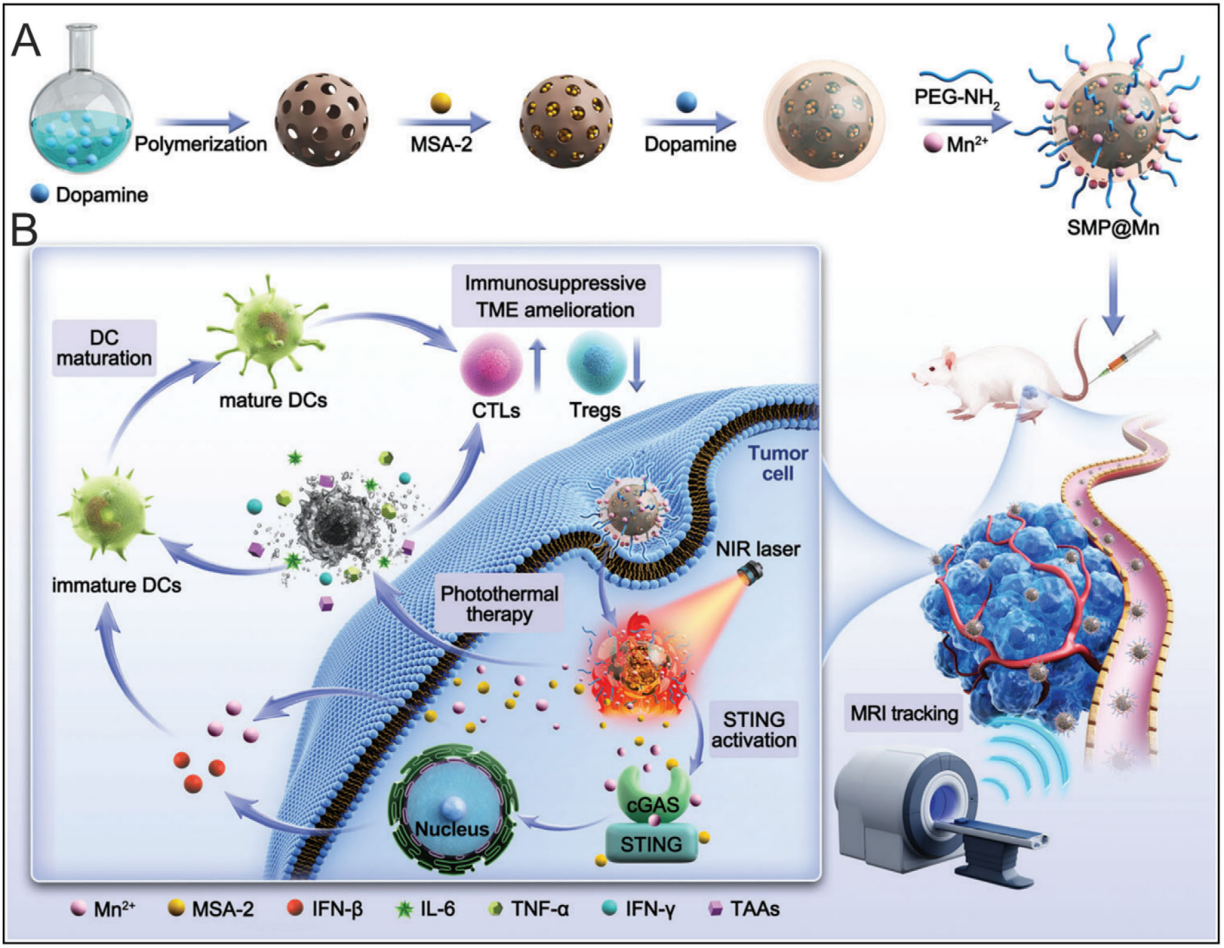
Schematic illustration of the multifunctional nanoplatform (SMP@Mn) combining PTT and STING pathway activation‐based immunotherapy. A) Preparation process and structure of SMP@Mn. B) Treatment procedure and mechanism of SMP@Mn against solid tumor by MRI‐guided PTT combined immunotherapy. Reproduced with permission. [114d] Copyright 2022, JohnWiley & Sons.

Alternatively, Zeng et al. recently demonstrated that combining PTT with dual STING agonists, MSA‐2 and Mn^2+^, could trigger robust systemic antitumor immunity in an orthotopic 4T1 murine breast cancer model (**Figure** **10**).^[^ ^114d^ ^]^ In this study, MSA‐2 was loaded into mesoporous polydopamine nanoparticles (MPDA) through π–π stacking interactions, after which dopamine was added to form a polydopamine coating. This film was then coordinated with Mn^2+^ ions and coated with PEG‐NH_2_ to improve nanoparticle stability and extend blood circulation, resulting in SMP@Mn nanoparticles.^[^ ^114d^ ^]^ Notably, this nanotherapeutic could be degraded in the slightly acidic TME, ensuring efficient payload release. Following systemic administration and NIR laser irradiation, SMP@Mn significantly activated the STING pathway, promoted DC maturation, and increased cytotoxic T‐cell infiltration at tumor sites. Compared to MSA‐2 monotherapy or SMP@Mn treatment without laser irradiation, the combination therapy demonstrated superior antitumor immunity and therapeutic efficacy in the orthotopic 4T1 breast cancer model.^[^ ^114d^ ^]^

Zhan et al. engineered a photoactivatable, thermal‐responsive liposomal platform, referred to as dNAc, for mild PTT‐augmented combinational chemodynamic‐immunotherapy.^[^ ^114e^ ^]^ The platform encapsulated the STING agonist cGAMP and ferrous sulfide (FeS_2_) nanoparticles within liposomes via passive loading, followed by surface modification with bromelain to facilitate extracellular matrix degradation. Notably, FeS_2_ nanoparticles served dual functions: as NIR‐II photothermal conversion agents for PTT and as catalysts for the Fenton reaction in chemodynamic therapy.^[^ ^114e^ ^]^ After intravenous injection and subsequent tumor accumulation at the tumor site, the tumors were exposed to NIR‐II light irradiation. The mild heat generated by FeS_2_ nanoparticles induced ICD and improved the Fenton reaction, which led to direct tumor cell killing and cGAS activation.^[^ ^114e^ ^]^ Furthermore, PTT‐mediated heat at the tumor sites facilitated the on‐target release of cGAMP from the thermo‐sensitive liposomes, further amplifying cGAS/STING signaling activation. Systemic administration with dNAc combined with NIR‐II photoactivation significantly decreased primary tumor growth, suppressed distant metastasis, and inhibited liver and lung metastasis in a murine model.^[^ ^114e^ ^]^ These results highlight the immense therapeutic potential of combining STING agonist‐based immunotherapy with PTT.

These results suggest that the combination of PTT with STING agonist can further improve the efficacy of PTT and substantially augment systemic antitumor immune activation, demonstrating significant potential for combinational therapy of STING agonist‐based immunotherapy and PTT.

#### 3.4.2 STING Agonists‐Based Nanotherapeutics Combined with PDT

In contrast to PTT, which induces tumor cell death through hyperthermia via photothermal conversion, PDT represents another clinically promising phototherapy paradigm for treating various tumors with high selectivity and minimal invasiveness.^[^ ^108^ ^]^ PDT leverages photosensitizers that generate cytotoxic ROS under localized tumor‐focused NIR light irradiation, thereby killing tumor cells and inducing an antitumor immune response.^[^ ^115^ ^]^ Although the contribution of PDT to both local and systemic antitumor immunity is inherently complex and not fully understood, numerous informative reviews have discussed the basic principles of PDT and its interplay with the immune system.^[^ ^108^, ^115^, ^116^ ^]^ Recent studies have shown that certain photosensitizers and PDT regimens can modulate the host immune system, such as inducing an ICD cascade, secreting pro‐inflammatory cytokines, stimulating the proliferation of NK cells,^[^ ^117^ ^]^ promoting antitumor immunity, and enhancing cancer immunotherapy sensitivity.

However, PDT‐induced antitumor immunity is often insufficient to control established tumors due to the intricate and diverse nature of the TME, which includes factors such as intrinsic immunosuppression, hypoxia, abnormal tumor vasculature, and other complicating elements.^[^ ^118^ ^]^ Additionally, PDT can sometimes induce immune suppression or tolerance. This arises from the widespread release of self‐antigens due to collateral damage to healthy cells, the secretion of immunosuppressive cytokines, and the oxidative modification of danger signals, all of which favor immune tolerance.^[^ ^118a^ ^]^ Thus, combining immunosimulators, particularly STING agonists, with PDT has been proposed as a promising strategy to counteract PDT's immunosuppressive limitations while enhancing the effectiveness of immunotherapy.

The main mechanistic aspects behind combining PDT with STING agonists are analogous to those discussed for PTT in Section 3.4.1. PDT induces ICD in irradiated tumor cells and increases the release of damaged nuclear or mitochondrial DNA (mtDNA) from dying tumor cells. This results in the presentation of more DNA antigens exposed on the surface of dying tumor cells, activating the cGAS‐STING pathway and sensitizing the tumor to immune responses. Notably, PDT also induces rapid neutrophil recruitment to the treatment site within minutes after irradiation, which helps to clear residual tumor cells and secrete pro‐inflammatory cytokines to further boost the immune responses. Additionally, PDT enhances the upregulation of heat shock proteins and other stress‐related proteins, promoting DCs activation and subsequent antigen presentation to T cells.

Nevertheless, PDT's efficacy is constrained by hypoxia and poor tissue penetration, particularly in deep‐seated or large tumors with abnormal tumor vasculature and low oxygen supply. In contrast, STING agonist‐based immunotherapy can circumvent these limitations by inducing robust systemic immune responses, effectively eliminating highly hypoxic tumors, and addressing the localized immune effects and tumor type constraints inherent to PDT. Despite progress in understanding the complementary mechanisms of these therapies, the precise ways in which PDT contributes to immune system activation and supports STING agonist‐mediated cancer immunotherapy remain extremely fragmentary. Further mechanistic studies are urgently needed to dissect these interactions, which will be crucial for advancing the clinical translation of these combination therapies for cancer treatments. The following section will summarize current findings on the interplay between PDT and STING agonists, focusing on their synergistic immunological effects and therapeutic potential.

**Figure 11:**
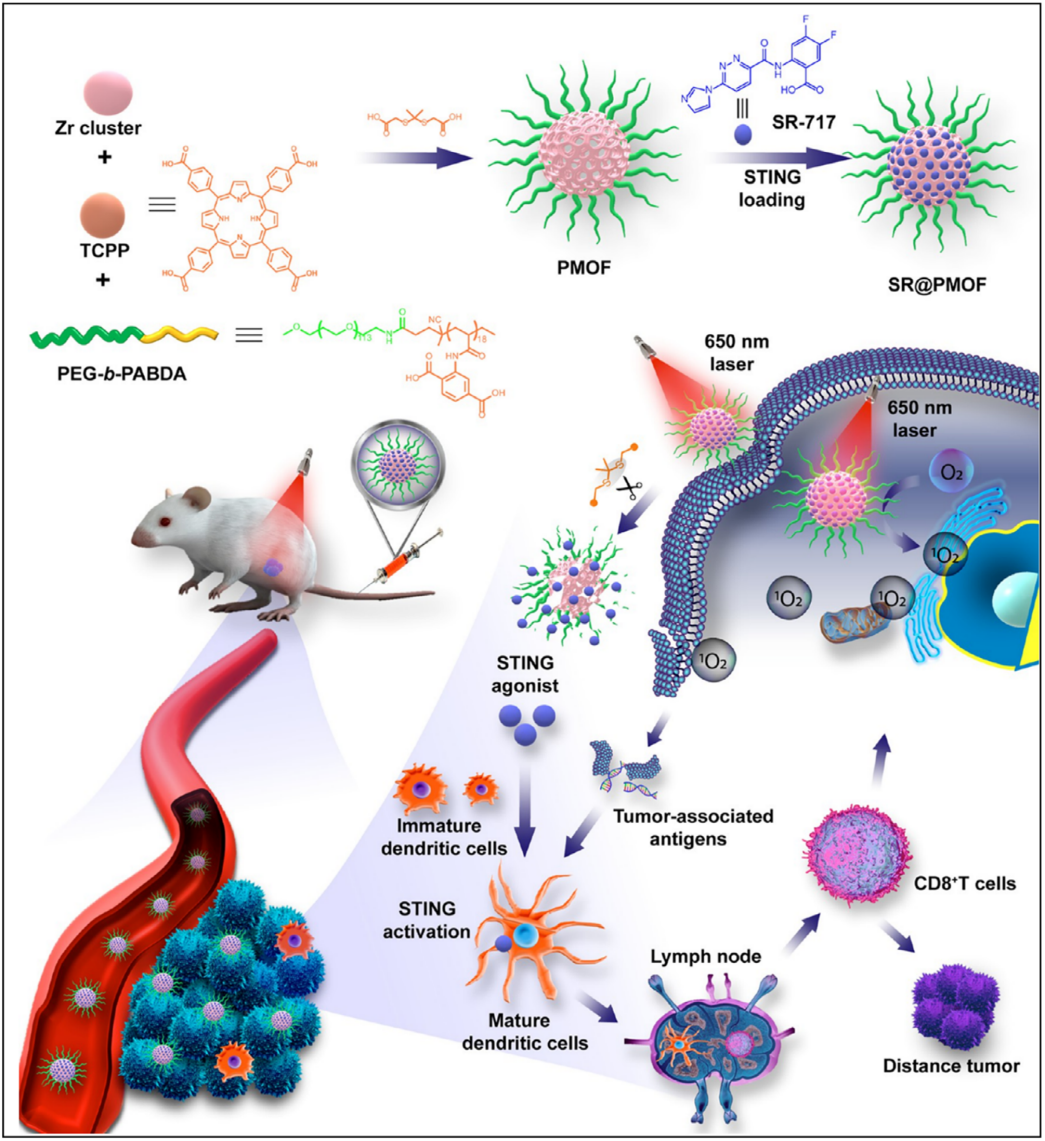
Scheme illustration of the preparation of SR‐717‐loaded oxidation‐responsive PMOF NPs and photodynamic‐immunotherapy of the tumors. The STING agonist SR‐717 was loaded by utilizing the porous structure of PMOF NPs and finally forming the SR@PMOF with PEG shells. After SR@PMOF accumulated in the tumor site by intravenous injection, the red light with the wavelength of 650 nm irradiated the tumor site to produce ROS and accelerated the release of STING agonist SR‐717 for antitumor immunity of enhanced STIGN activation. Reproduced with permission.^[^ ^119a^ ^]^ Copyright 2022, American Chemical Society.

Selected examples demonstrate how rational nanotechnology design can integrate PDT and STING agonists‐based immunotherapy into a single nanosystem to achieve synergistic therapeutic effects.^[^ ^117^, ^119^ ^]^ For instance, Ge et al. developed an oxidation‐responsive polymeric metal‐organic framework (PMOF) nanoparticles for combining PDT with STING agonist‐mediated cancer immunotherapy (**Figure** **11**).^[^ ^119a^ ^]^ These nanoparticles were fabricated by co‐assembling PEG‐b‐PABDA block copolymer (comprising PEG and 1,4‐bezenedicarboxylic acid bearing polyacrylamide (PABDA)), the photosensitizer meso‐tetra(carboxyphenyl)‐porphyrin (TCPP), an oxidation‐responsive thioketal diacetic acid group, and zirconyl chloride (ZrOCl_2_·8H_2_O).^[^ ^119a^ ^]^ The produced PMOF nanoparticles (≈50 nm in diameter) featured a highly porous structure, which was employed to load the STING agonist SR‐717 to form the final SR@PMOF NPs. Upon NIR irradiation, TCPP‐mediated PDT generated ROS, which cleaved the thioketal bonds, facilitating the rapid release of SR‐717. This triggered robust activation of STING signaling and enhanced antitumor immunity.^[^ ^119a^ ^]^ Intravenous injection of SR@PMOF NPs in mice reversed the immunosuppressive TME and effectively controlled both primary and distant tumors, demonstrating the significant synergy between PDT‐induced immune activation and STING agonist‐based immunotherapy in cancer treatment.^[^ ^119a^ ^]^

Ren et al. designed a metal‐organic framework‐based nanoagonist (DZ@A7) with NIR/TME‐responsive decomposition properties, enabling tumor‐specific STING activation for enhanced photodynamic metalloimmunotherapy.^[^ ^119b^ ^]^ The nanoparticles were fabricated by mixing Zn^2+^ and 2‐methylimidazole to form ZIF‐8, a framework with a large surface and high porosity. These nanoparticles were then loaded with the hypoxic drug banoxantrone dihydrochloride (AQ4N) and photosensitizer IR780 through physical adsorption, followed by a polydopamine coating to obtain DZ@A7.^[^ ^119b^ ^]^ DZ@A7 generated mitochondria‐targeted ROS under NIR irradiation, which promoted the release of mtDNA to activate the cGAS‐STING pathway. Additionally, it facilitated the activation of AQ4N. Synchronously, NIR‐induced Zn^2+^ release further enhanced cGAS enzymatic activity via metalloimmune effects.^[^ ^119b^ ^]^ Notably, the combined cascade STING activation by DZ@A7 under NIR irradiation promoted the maturation of DCs, enhanced tumor infiltration of CTLs , and significantly attenuated both primary and metastatic 4T1 tumor growth compared to monotherapies.^[^ ^119b^ ^]^ These findings highlight the substantial potential of NIR‐driven STING activation and synergistic PDT‐metalloimmunotherapy in nano‐based cancer treatment.

Wu et al. developed an innovative PDT‐metalloimmunotherapy delivery system using porphyrin‐based photosensitizers with structural modifications to enhance Mn^2+^ intracellular transport.^[^ ^119c^ ^]^ Exploiting hematoporphyrin's metal complexation properties, they synthesized various manganese porphyrins (photo‐STING agonist, PSA) and evaluated their ability to activate the cGAS‐STING pathway in macrophages. The optimal PSA was encapsulated in a protein complex of human hemoglobin and albumin, with perfluorohexane as an ultrasound‐responsive agent to rupture the external membrane and expose hemoglobin binding sites for spatiotemporal uptake by TAMs via CD163 receptors.^[^ ^119c^ ^]^ The nanoparticles (PP‐GA) were coated with a red blood cell membrane to extend blood circulation and improve tumor targeting. Upon intravenous administration, the NPs accumulated at the tumor site, where ultrasonication triggered PSA release for TAMs uptake. PSA generated ROS via PDT under light irradiation, causing mtDNA damage and activating the cGAS‐STING pathway in macrophages. This promoted TAM polarization, increased pro‐inflammatory cytokine secretion, and enhanced DC maturation and T‐cell infiltration, thereby stimulating a potent anti‐cancer immune response.^[^ ^119c^ ^]^ This work demonstrates the synergistic effect of photo‐STING agonists and fractured mtDNA in repolarizing TAMs for enhanced cancer immunotherapy.

Huang et al. developed a rationally designed polymersome‐grafted type I photosensitizer (PNBS) for loading STING agonists, creating nanovesicles (termed PNBS/DiABZI) for enhanced cancer immunotherapy.^[^ ^117^ ^]^ The PNBS could orient type I photosensitizers (capable of generating ROS via oxygen‐independent electron transfer) towards H‐aggregation and intersystem crossing, extending the lowest‐energy triplet excited states and resulting in a ≈3‐fold increase in ROS yield for PDT.^[^ ^117^ ^]^ The STING agonist diABZI was loaded via the nanoprecipitation method. Both in vitro and in vivo experiments showed that PDT with PNBS/DiABZI effectively destroyed hypoxic tumors and facilitated the proliferation of CTLs and NK cells, overcoming resistance linked to reduced NK cell proliferation observed with STING agonist monotherapy.^[^ ^117^ ^]^ Remarkably, a single intravenous dose of PNBS/diABZI nanovesicles completely eliminated orthotopic mammary tumors in mice under laser irradiation, induced long‐lasting antitumor immune memory, and conferred resistance to subcutaneous or intravenous tumor rechallenge with 4T1 cells. In contrast, PDT or STING immunotherapy alone failed to achieve similar outcomes.^[^ ^117^ ^]^

In contrast to the aforementioned nanoplatforms that coencapsulate photosensitizers and STING agonists for synchronized delivery, the following study adopts a distinct strategy by physically mixing separate nanoparticles, each loaded with a single therapeutic agent, to achieve synergistic immunotherapeutic effects. For example, Xu et al. developed a dual‐nanoparticle system (MP@PPS NPs) by combining ROS‐responsive poly(propylene sulfide) nanoparticles (PPS NPs) separately loaded with the STING agonist MSA‐2 or the photosensitizer PPa, achieving a synergistic photo‐immunotherapeutic response in the B16F10 melanoma model.^[^ ^120^ ^]^ PPS NPs were synthesized via emulsion polymerization of poly(propylene sulfide) and Pluronic F127. MSA‐2 and PPa were then conjugated to the PPS NPs via click chemistry to generate MSA@PPS NPs and PPa@PPS NPs, respectively. These two nanoparticle formulations were subsequently mixed to form the final MP@PPS NPs. Following intravenous injection and laser irradiation, this formulation significantly promoted DC maturation, enhanced intratumoral infiltration of CD8+ CTLs and NK cells, and increased TNF‐α and IFN‐β while reducing immunosuppressive IL‐10 levels. Compared to monotherapies or the soluble combination of MSA‐2 and PPa, MP@PPS NPs elicited markedly stronger antitumor immune responses. These results highlight the potent synergy achieved through the combination of PDT and STING activation.^[^ ^120^ ^]^

Taken together, these findings demonstrate the potent synergy of PDT and STING agonists in nanosystems, offering a transformative strategy to overcome tumor immune suppression, enhance systemic antitumor immunity, and achieve superior therapeutic outcomes in cancer immunotherapy.

### 3.5 STING Agonists‐Based Nanotherapeutics Combined with SDT

SDT is a clinically promising tumor treatment modality characterized by noninvasiveness, high spatiotemporal control, and deep tissue penetration.^[^ ^121^ ^]^ SDT utilizes ultrasonic waves to activate sonosensitizers, generating ROS, cavitation, gas bubbles, and hyperthermia for cancer therapy.^[^ ^121c^ ^]^ Beyond causing oxidative stress‐induced tumor cell death, SDT can also trigger ICD, thereby sensitizing tumors to immunotherapy.^[^ ^122^ ^]^ However, its therapeutic efficacy is often compromised by the hypoxic tumor TME, which limits ROS generation.^[^ ^123^ ^]^ SDT‐induced ICD alone is typically insufficient to reverse the inherently immunosuppressive TME or elicit robust systemic antitumor immunity. Therefore, combining SDT with immunostimulants, such as STING agonists, has emerged as a promising strategy to synergistically augment antitumor immunity.

The rationale for combining STING agonists with SDT is multifaceted. First, STING agonists robustly activate both innate and adaptive immunity. Reciprocally, SDT enhances cellular membrane permeability through ultrasound‐induced effects, facilitating the intracellular transport of poorly internalized STING agonists and improving their delivery to deeper tumor tissues. This complementary mechanism helps overcome the delivery limitations of STING agonists and amplifies local and systemic antitumor immune responses. Furthermore, SDT enables precise, controlled activation of therapeutic agents, mitigating the continuous pharmacological activation of STING agonists in normal tissues. This spatiotemporal control reduces immune‐related adverse events and optimizes therapeutic efficacy, offering a safer and more effective cancer treatment strategy.

Many preclinical studies have highlighted the potential of combining SDT with STING agonists to elicit sustained and distant antitumor immune responses.^[^ ^124^ ^]^ An exemplary study by Huang et al. developed a nanoparticle system, FA‐ICG&MnO_x_@HSA, comprising human serum albumin, the sonosensitizer indocyanine green (ICG), and the STING agonist manganese oxide (MnO_x_). This system demonstrated synergistic sono‐immunotherapeutic effects in organoid and mouse tumor models.^[^ ^124a^ ^]^ To combat hypoxia, a critical limitation of SDT that impairs ROS generation, Zhang and colleagues developed a liposomal PFH nanodroplet encapsulating the STING agonist DMXAA and the sonosensitizer IR‐780, referred to as IDP.^[^ ^124b^ ^]^ By loading O_2_ gas into the PFH core, the O_2_‐filled nanodroplet (IDP@O_2_) was engineered to respond to ultrasound irradiation. The released O_2_ significantly enhanced the ROS production and boosted the efficacy of SDT. As a result, IDP@O_2_ exhibited potent synergistic antitumor effects through the combination of SDT and STING activation, inducing long‐term immunological memory and effectively inhibiting both primary and distant tumor growth in a TNBC model.^[^ ^124b^ ^]^

The strategic design of nanomedicines employing polymers as nanocarriers offers a promising approach to amplify both localized and systemic therapeutic effects of STING agonists. Polymer‐STING agonist conjugates have emerged as effective building blocks for constructing nanoparticulate delivery systems. For example, semiconducting polymers with efficient sonodynamic properties have been used as nanocarriers, covalently conjugated with STING agonists to achieve synergistic sono‐immunotherapy.^[^ ^124c,d^ ^]^ In a notable study, Pu et al. developed a redox‐responsive amphiphilic semiconducting polymer prodrug capable of selectively activating the STING pathway through glutathione (GSH)‐mediated release of MSA‐2 in the TME.^[^ ^124c^ ^]^ The STING agonist (MSA‐2) was conjugated to the PEG side chains on a semiconducting polymer backbone (poly[2,7‐(9,9‐dioctylfluorene)‐alt‐4,7‐bis (thiophen‐2‐yl) benzo‐2,1,3‐thiadiazole]) (PFODBT) via a GSH‐activatable disulfide linker. This polymer‐drug conjugate self‐assembled into nanoparticles with a diameter of ≈28.2 nm, termed PSPA. Following intravenous administration, PSPA accumulated in tumor sites and generated ROS under sono‐irradiation, inducing ICD and stimulating antitumor immune responses. Simultaneously, the high GSH concentrations in the TME triggered disulfide bond cleavage, releasing MSA‐2 and activating the STING pathway, thereby further amplifying tumor immunogenicity.^[^ ^124c^ ^]^ In a bilateral flank 4T1 breast cancer model, PSPA demonstrated superior therapeutic efficacy by significantly improving survival and reducing tumor recurrence compared to either sono‐irradiation or MSA‐2 treatment alone. Follow‐up rechallenge experiments revealed that mice treated with PSPA under sono‐irradiation generated durable immunological memory and long‐term protective immunity, highlighting the potential of this strategy to prevent tumor relapse.^[^ ^124c^ ^]^

**Figure 12:**
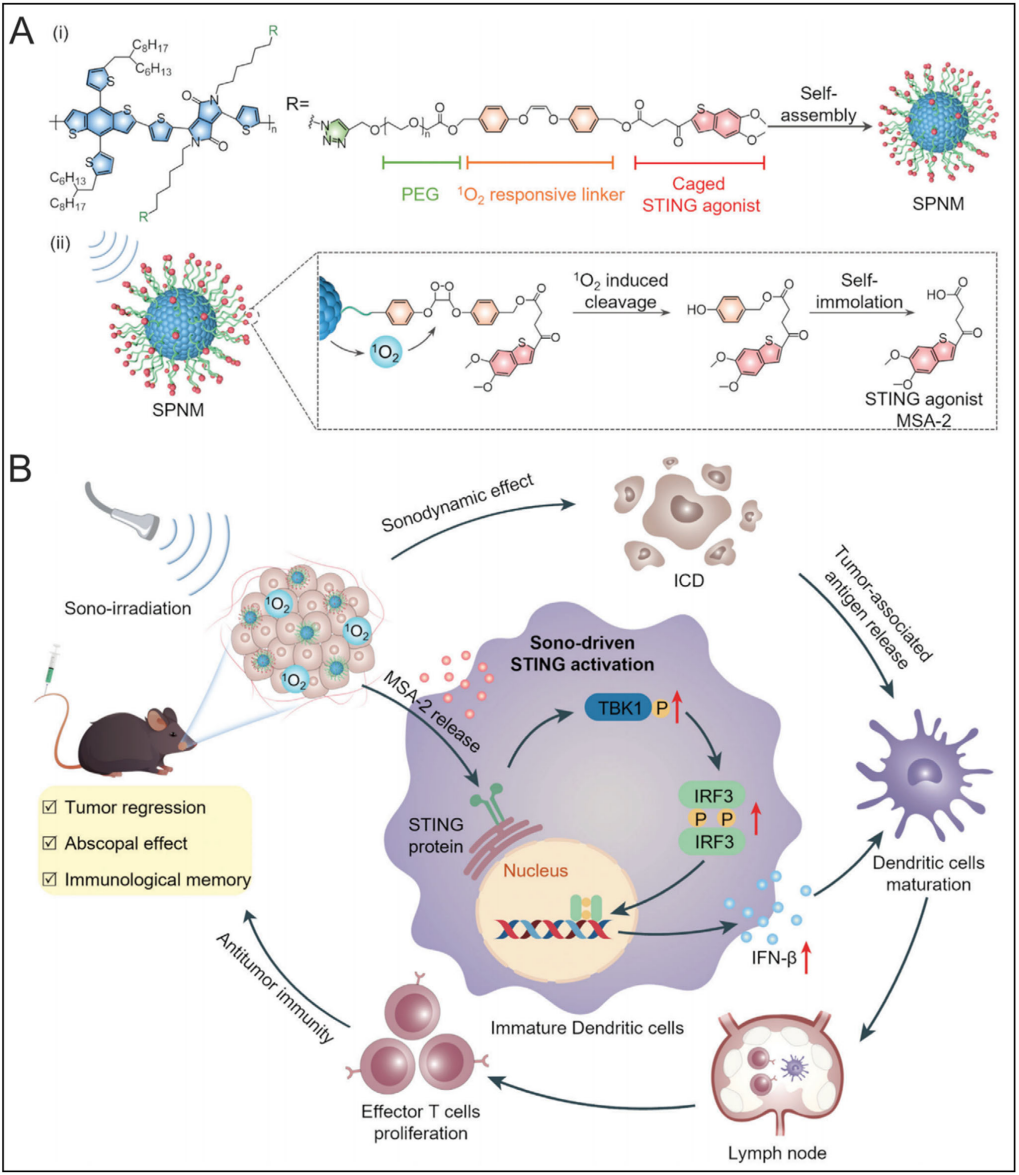
Schematic illustration of SPNM‐mediated sono‐driven STING activation for sono‐immunotherapy of HNSCC. (A) i) Chemical structure and preparation of SPNM. ii) Sonodynamic scission of the diphenoxyethene bond releases MSA‐2 from SPNM. (B) Proposed mechanism of the SPNM‐mediated sonodynamic effect to induce STING activation and ICD, stimulate cytokine secretion, trigger DC maturation, and promote effector T cell proliferation for antitumor immunity. Reproduced with permission.^[^ ^124d^ ^]^ Copyright 2023, John Wiley & Sons.

Analogous to the efforts mentioned above, Zhen et al. developed a semiconducting polymeric nanoagonist (SPNM) for in situ sono‐driven STING activation to enhance immunotherapy (**Figure** **12**).^[^ ^124d^ ^]^ A ^1^O_2_‐cleavable linker ((Z)‐((ethene‐1,2‐diylbis(oxy)) bis (4,1‐phenylene)) dimethanol) (EOPD), was synthesized to respond to high ROS levels in the TME and release the active drug. To introduce amphiphilicity, EOPD was reacted with alkyne‐PEG‐COOH, forming a PEG‐EOPD conjugate, which was further linked to the STING agonist MSA‐2 to yield a PEG‐EOPDM conjugate. The sonodynamic semiconducting polymer (SPM) was synthesized using palladium‐catalyzed Still polymerization and subsequently conjugated to PEG‐EOPDM via click chemistry, yielding the final amphiphilic polymer.^[^ ^124d^ ^]^ This polymer self‐assembled into nanoparticles (SPNM) with a diameter of ∼34 nm. In orthotopic murine models of HNSCC, therapeutic analyses showed that SPNM, under sono‐irradiation, exhibited significantly enhanced efficacy compared to control nanoparticles lacking either the EOPD linker (SPND) or the STING agonist (SPNC). These results highlight the synergistic potential of SDT and STING activation for advancing cancer immunotherapy.^[^ ^124d^ ^]^

These efforts collectively exemplify the synergistic effects of combining SDT with STING agonists in activating antitumor immunity. By leveraging complementary mechanisms, this combination strategy effectively overcomes the immunosuppressive tumor TME while significantly enhancing both localized and systemic antitumor immune responses, paving the way for innovative approaches in advanced cancer immunotherapy.

### 3.6 STING Agonists‐Based Nanotherapeutics Combined with Targeted Therapy

Targeted therapy employs drugs that selectively target and precisely modulate dysregulated signaling pathways in cancer cells, effectively inhibiting tumor growth while minimizing toxicity to normal tissues. Compared to conventional chemotherapy, targeted therapies generally elicit more durable responses with fewer adverse effects. However, resistance to single‐agent targeted therapies remains a major clinical challenge, particularly in advanced cancers.^[^ ^125^ ^]^ In this context, combination therapies that simultaneously target multiple oncogenic and immunoregulatory pathways have emerged as a promising approach to enhance therapeutic durability and overcome drug resistance.

A particularly compelling therapeutic synergy exists between STING agonists and DNA damage response (DDR)‐targeting agents, such as Rad3‐related protein inhibitors^[^ ^32e^ ^]^ and poly(ADP‐ribose) polymerase (PARP) inhibitors.^[^ ^126^ ^]^ These agents induce DNA damage, accumulation, leading to the generation of neoantigens and subsequent STING pathway activation, thereby enhancing tumor immunogenicity. PARP inhibitors, in particular, have demonstrated synthetic lethality in BRCA‐deficient cancers, including breast and ovarian tumors harboring loss‐of‐function mutations in homologous recombination pathway genes, primarily BRCA1 and BRCA2.^[^ ^126^ ^]^ This dual mechanism, i.e., DNA damage induction and innate immune activation via STING, presents an opportunity for enhanced therapeutic efficacy in BRCA‐deficient tumors when STING agonists and DDR‐targeting agents are used in combination.

The interplay between PARP inhibition and STING activation has been extensively explored in preclinical studies. For instance, Zhao et al. demonstrated that STING agonists could sensitize BRCA1‐deficient breast cancer cells to PARP inhibitors by reprogramming TAMs from an immunosuppressive M2‐like phenotype to a pro‐inflammatory M1‐like phenotype, thereby restoring the synthetic lethal response to PARP inhibition.^[^ ^127^ ^]^ Furthermore, systemic administration of the STING agonist DMXAA, combined with the PARP inhibitor Olaparib, induced strong antitumor immunity and significantly improved therapeutic outcomes in BRCA1‐deficient mouse models of breast cancer.^[^ ^127^ ^]^ These findings provide compelling evidence that STING pathway activation can help overcome resistance to DDR‐targeting therapies, offering a rationale for clinical translation of STING‐PARP combination therapies.

Despite being a promising approach to overcome resistance mechanisms in BRCA‐deficient tumors, challenges related to systemic delivery, off‐target toxicity, and acquired treatment resistance remain barriers to clinical translation. Emerging nanotechnologies offer innovative solutions to these obstacles, offering precisely controlled, tumor‐specific, and immune‐responsive delivery platforms that can maximize therapeutic efficacy while minimizing toxicity. Moving forward, integrating advanced nanocarrier designs, personalized medicine strategies, and combination immunotherapy will be essential to bridge the gap between preclinical success and clinical application, ultimately setting the stage for more potent and long‐term cancer therapies.

## 4 Conclusion and Future Perspectives

The cGAS‐STING pathway, recognized as a promising therapeutic target, has been gaining increasing attention in tumor biology and cancer immunotherapy due to its critical role in orchestrating antitumor immune responses. Although STING agonists have demonstrated substantial therapeutic potential, their clinical application remains hindered by drug delivery challenges, pharmacological limitations, and translational bottlenecks.

The rapid advancement of nanotechnology in drug delivery offers numerous opportunities to enhance the efficacy of cGAS‐STING agonist‐based therapeutics, leading to significant progress in recent years. Given these advancements, it is both timely and essential to comprehensively review the latest developments in STING agonist‐driven nanotherapeutics. This review highlights the recent advances in this field, with a focus on the brief development of cGAS‐STING agonist‐based nanotherapeutics, as well as underlying molecular mechanisms, rationale, and applications of STING agonist‐based combination therapies.

Despite the rapid progress and clinical potential of cGAS‐STING agonists, none have yet achieved market approval, despite their entry into clinical trials as monotherapies and in combination with standard‐of‐care treatments. Several key challenges must be addressed to facilitate the effective pharmaceutical development of cGAS‐STING agonists. A major bottleneck is the druggability of STING agonists. While numerous chemical compounds targeting this pathway have been identified, the development of highly druggable and structurally optimized STING agonists remains a critical unmet need. Another major challenge in the clinical translation of cGAS‐STING agonists is treatment‐related toxicities, which continue to raise significant safety concerns. Notably, systemic administration of STING agonists, regardless of their type or formulation, has been associated with dose‐limiting toxicity and a narrow therapeutic window. Therefore, expanding this therapeutic window by mitigating inflammatory side effects will be essential for advancing their clinical utility.

Beyond these pharmacological hurdles, counter‐regulatory mechanisms induced by STING activation present additional challenges. STING pathway activation can trigger immunosuppressive feedback loops, including the production of IDO1, upregulation of PD‐L1, and other immune checkpoint molecules, and recruitment of immunosuppressive MDSCs, ultimately dampening antitumor immunity. These responses can impair DC function, suppress cytotoxic T lymphocyte activity, and re‐establish an immunosuppressive tumor microenvironment, thereby attenuating the therapeutic efficacy of STING agonists. In certain contexts, STING‐induced chronic inflammation may even contribute to acquired resistance and paradoxical tumor progression by fostering an environment conducive to immune escape and stromal remodeling. However, the biological underpinnings and temporal dynamics of these resistance mechanisms remain poorly understood. For example, it is unclear whether different tumor types or immune contexts exhibit distinct thresholds of STING activation that shift its role from immune stimulation to immune suppression. Further complicating this landscape is the lack of robust predictive biomarkers to guide the design of combination regimens or identify patients who are most likely to benefit from STING‐based therapies. Therefore, identifying and validating context‐specific biomarkers, such as STING expression levels, downstream cytokine profiles, immune checkpoint signatures, and myeloid cell phenotypes, is urgently needed to facilitate rational patient stratification and personalized therapeutic interventions. Addressing these knowledge gaps will be pivotal for converting transient immunostimulatory effects into durable clinical responses.

On the translational front, nanomedicine faces significant barriers, including challenges in large‐scale manufacturing, ensuring batch‐to‐batch consistency, and maintaining long‐term stability of nanoformulations. In addition, regulatory uncertainty and the complexity of clinical trial design for nano‐immunotherapeutics further hinder their translation from bench to bedside. Harmonized regulatory frameworks and the implementation of quality‐by‐design (QbD) principles in manufacturing are urgently needed to ensure reproducibility, safety, and scalability.

Despite these challenges, several emerging technologies hold immense promise for accelerating the clinical translation of STING agonists in the foreseeable future. First, artificial intelligence (AI) and machine learning can revolutionize high‐throughput drug screening, enabling the discovery of novel drug‐like STING agonists with improved pharmacokinetics and bioavailability. Second, advances in nanotechnology, pharmaceutical, chemical engineering, and biomedical engineering are paving the way for tumor‐selective delivery strategies, allowing for precise STING activation in cancer cells while minimizing systemic inflammatory toxicity. Particularly, stimuli‐responsive and biomimetic nano‐delivery systems are being developed to enhance tumor targeting, control drug release, and optimize immunomodulation. Additionally, interdisciplinary research networks integrating AI, spatial transcriptomics, single‐cell sequencing, proteomics, and metabolomics should be established to elucidate the intricate interplay between STING agonists and the tumor immune microenvironment. These efforts should prioritize identifying mechanisms underlying therapeutic resistance, deciphering tumor‐specific immune responses, and optimizing combination immunotherapy strategies. Specifically, preclinical models with spatial and temporal resolution of STING signaling, as well as integrative multi‐omics approaches, will be essential to map resistance pathways and uncover new targets for combination strategies. In this regard, current research is actively exploring nano‐DDSs capable of co‐delivering STING agonists alongside agents targeting complementary immunological and oncogenic pathways. Insights from preclinical models and ongoing clinical trials will be instrumental in closing these knowledge gaps. Furthermore, proteomic approaches hold promise for identifying novel predictive biomarkers, facilitating patient stratification, and overcoming resistance mechanisms, ultimately maximizing the therapeutic potential of STING agonists.

As knowledge in this field continues to advance, our understanding of the immunopharmacological mechanisms of the cGAS‐STING pathway, along with the pharmacokinetic and pharmacodynamic properties of STING agonists and their corresponding drug delivery technologies, will become increasingly refined. Continued progress in chemical and biomolecular engineering will be critical to optimizing STING pathway activation, while advances in nanotechnology‐driven drug delivery will be essential for overcoming current translational challenges. Realizing the clinical potential of STING agonists will require sustained interdisciplinary collaboration across drug design, delivery optimization, immune profiling, and regulatory strategies. These coordinated efforts are essential to unlock their full promise as next‐generation immunotherapeutics and enable more effective and durable cancer treatments.

## Conflict of Interest

The authors declare no conflict of interest.
